# Tumor microenvironment responsive nano-herb and CRISPR delivery system for synergistic chemotherapy and immunotherapy

**DOI:** 10.1186/s12951-024-02571-9

**Published:** 2024-06-19

**Authors:** Yuanyuan Jia, Yuhui Yao, Lingyao Fan, Qiqing Huang, Guohao Wei, Peiliang Shen, Jia Sun, Gaoshuang Zhu, Zhaorui Sun, Chuandong Zhu, Xin Han

**Affiliations:** 1 The Second Affiliated Hospital of Nanjing University of Chinese Medicine, Jiangsu Collaborative Innovation Center of Chinese Medicinal Resources Industrialization, School of Medicine, Nanjing University of Chinese Medicine, Nanjing, 210023 China; 2 Department of Oncology, The Second Hospital of Nanjing, Nanjing University of Chinese Medicine, Nanjing, 210003 China; 3 Department of Emergency Medicine, Jinling Clinical Medical College, Nanjing University of Chinese Medicine, Nanjing, 210002 China

**Keywords:** Doxorubicin (DOX), Isoliquiritigenin (ISL), CRISPR-Cas 9, Protein tyrosine phosphatase non-receptor type 2 (PTPN2), Immunotherapy, Synergistic therapy

## Abstract

Chemoresistance remains a significant challenge for effective breast cancer treatment which leads to cancer recurrence. CRISPR-directed gene editing becomes a powerful tool to reduce chemoresistance by reprogramming the tumor microenvironment. Previous research has revealed that Chinese herbal extracts have significant potential to overcome tumor chemoresistance. However, the therapeutic efficacy is often limited due to their poor tumor targeting and in vivo durability. Here we have developed a tumor microenvironment responsive nanoplatform (H-MnO_2_(ISL + DOX)-PTPN2@HA, M(I + D)PH) for nano-herb and CRISPR codelivery to reduce chemoresistance. Synergistic tumor inhibitory effects were achieved by the treatment of isoliquiritigenin (ISL) with doxorubicin (DOX), which were enhanced by CRISPR-based gene editing to target protein tyrosine phosphatase non-receptor type 2 (PTPN2) to initiate long-term immunotherapy. Efficient PTPN2 depletion was observed after treatment with M(I + D)PH nanoparticles, which resulted in the recruitment of intratumoral infiltrating lymphocytes and an increase of proinflammatory cytokines in the tumor tissue. Overall, our nanoparticle platform provides a diverse technique for accomplishing synergistic chemotherapy and immunotherapy, which offers an effective treatment alternative for malignant neoplasms.

**Graphical Abstract:**

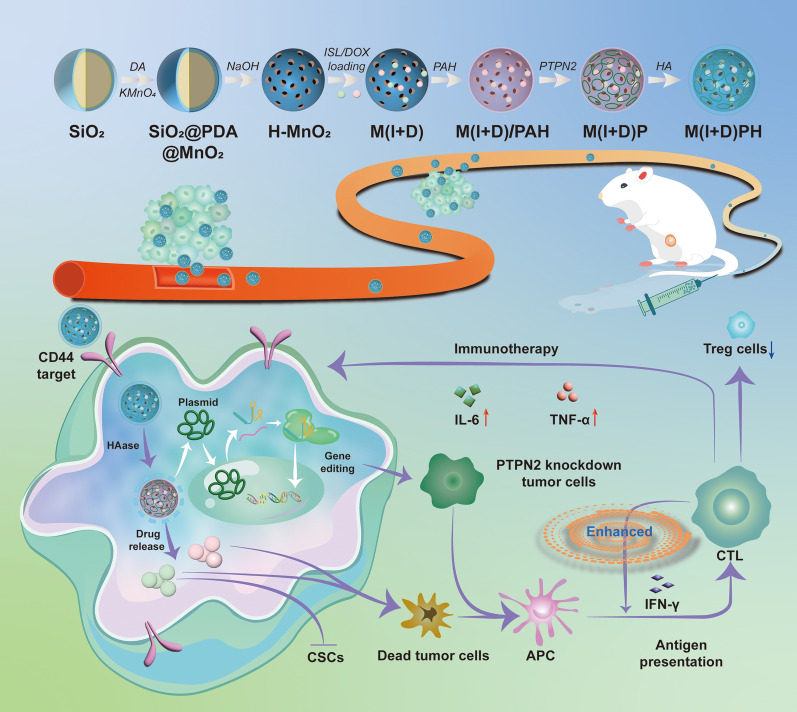

**Supplementary Information:**

The online version contains supplementary material available at 10.1186/s12951-024-02571-9.

## Introduction

Breast cancer (BC) has become a major threat to women's health worldwide, due to its early metastasis, with high recurrence rate and poor prognosis [1].At present, clinical treatment mainly includes surgery, chemotherapy or radiotherapy, immunotherapy and endocrine therapy [2–4]. However, huge challenges are still needed to be addressed such as poor tumor-targeting, side effects and chemo-resistance [5, 6]. For traditional chemotherapy drugs, chemoresistance make it difficult to achieve expected treatment effect [7]. Doxorubicin (DOX), a typical anti-cancer drug, is widely used in cancer treatment due to its broad-spectrum antitumor effect with the mechanisms of inducing tumor cells death by inhibition of topoisomerase II, intercalation of DNA, and production of free radicals [8]. But in recent years, the use of it has been increasingly limited due to severe side effects [9, 10]. Moreover, cancer cells usually develop resistance to its anti-tumor activity, such as the characteristics of cancer stem cells, which accounts for chemotherapy failure in cancer patients [11].

Increasing evidence has demonstrated that phytochemicals can be considered as potential agents for coadministration with chemotherapeutic agents in cancer therapy [12, 13]. Chinese traditional herbs play an auxiliary anti-cancer role by inducing cell apoptosis, enhancing immune system function, and reversing multiple drug resistance [14–16]. The anti-tumor effects of natural products, especially Chinese herbal extracts, drive increasing attention. Isoliquiritigenin (ISL), is a natural chalcone extracted from licorice, belonging to the flavonoids, and pertain to phytoestrogens [17, 18], which exerts anti-tumor effects through many mechanisms. In particular, ISL has an inhibitory effect on cancer stem cells [19–21], and the characteristics of cancer stem cells is closely related to cancer drug resistance. However, ISL has weak solubility, which has restricted its use. Drug delivery nanocarriers can deliver drugs to targeted cells and tissues, with the advantages of multidrug delivery capacity and reduced drug side effects [22, 23]. Hollow manganese dioxide (H-MnO_2_, M) is an excellent nanocarrier for drug delivery, with the advantages of low toxicity, high loading capacities. Moreover, it has ultrasensitive PH/GSH-triggered controllable drug release ability responsive to tumor microenvironment (TME) [24, 25].

The CRISPR-Cas9 technology has attracted great interest in cancer therapy applications with the advantages of high efficiency and easy operation, which can use sgRNA to target genome sequence and Cas9 protease to effectively cut the target gene [26–29]. Protein tyrosine phosphatase non-receptor type 2 (PTPN2) is considered to be a new target for tumor immunotherapy, and deletion of PTPN2 in tumor cells can strengthen immune response by enhancing interferon-γ (IFN-γ)-mediated effects on antigen presentation [30–33]. Moreover, PTPN2 also can influence T cell recruitment and T cell function [34–36]. Thus, targeting PTPN2 by CRISPR technology could be an effective method to enhance caner immunity effects to inhibit tumor recurrence and metastasis.

**Scheme 1:**
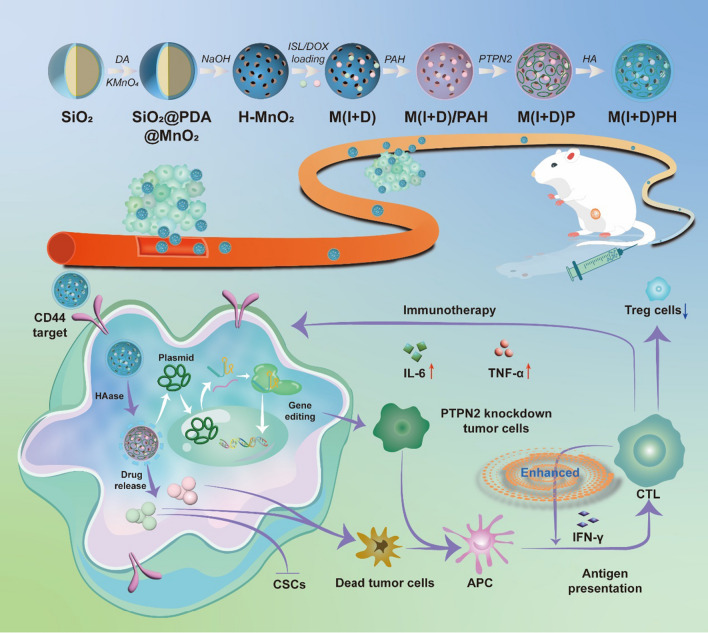
Design of M(I + D)PH nanocomposite and its antitumor therapeutic function. Schematic diagram of the preparation routes of M(I + D)PH nanoparticle and the synergistic chemotherapy and immunotherapy in vivo

Herein, we designed a novel nanoplatform M(I + D)PH (H-MnO_2_(ISL + DOX)-PTPN2@HA, M(I + D)PH) to realize precise drug delivery and gene editing with synergistic chemotherapy and immunotherapy. After the nanocomposite enter the blood vessels, it enriched towards the tumor site due to the enhanced permeability and retention (EPR) effect. Then, it entered the cell by the specific binding of the HA and CD44 receptors. The drugs were released in the state of high GSH in in cytosol of cancer cells. ISL inhibited cancer stem cells and cooperated with DOX to kill tumor cells. Cells underwent gene editing to target PTPN2, which can greatly activate the antigen presentation effects and boost tumor immunity in vivo. After the synergistic chemotherapy and cancer immunity, maximized anti-tumor effect were achieved (Scheme 1).

## Results

### ISL enhances chemosensitivity of DOX in vitro

**Fig. 1:**
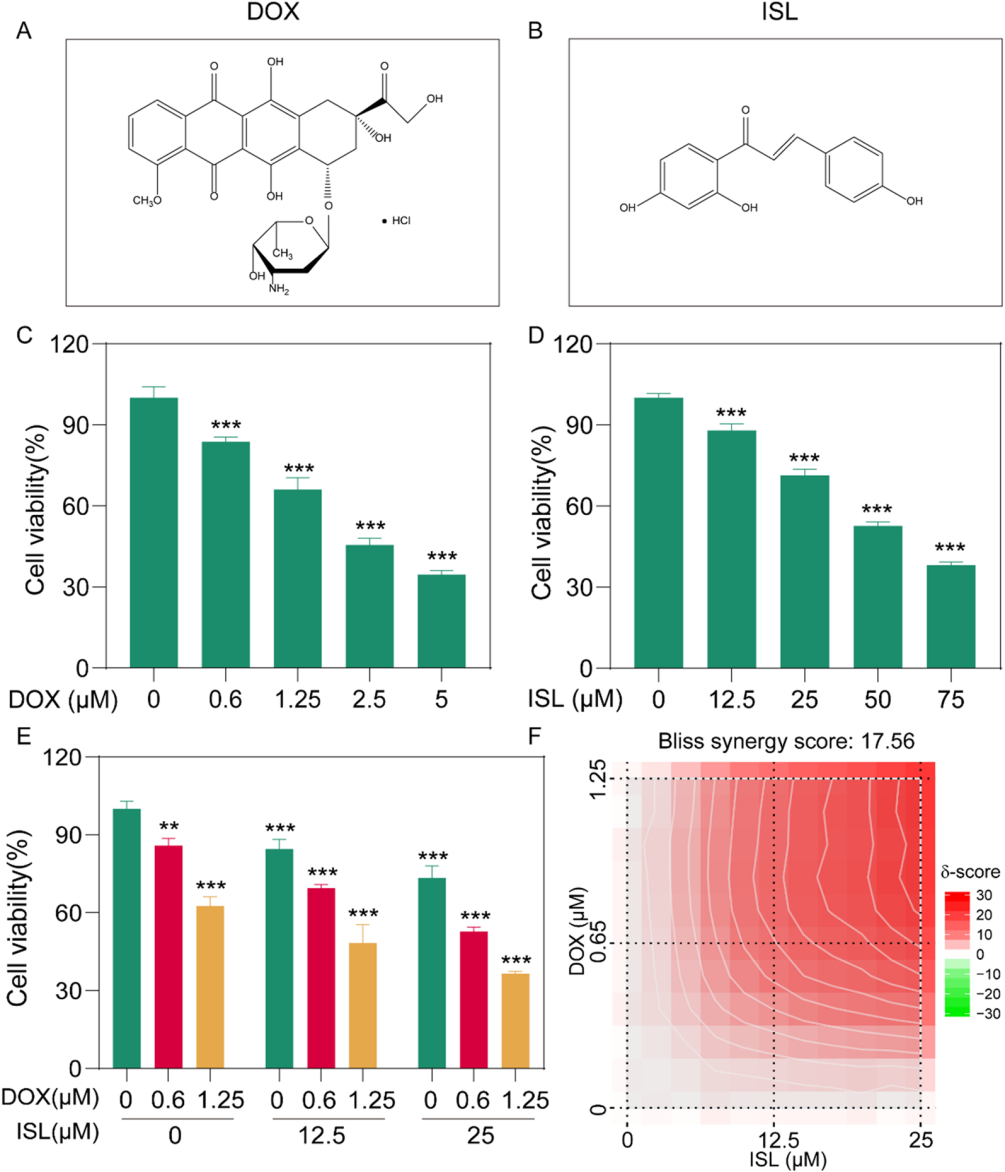
Synergistic inhibitory effects of DOX and ISL on cell viability of 4T1 cells. **A–B** Chemical formula of DOX (**A**) and ISL (**B**). **C** Cell viability of 4T1 cells treatment of different concentrations of DOX (n = 3). **D** Cell viability of 4T1 cells treatment of different concentrations of ISL (n = 3). **E** 4T1 cells treated with DOX of different concentrations in combination of ISL (n = 3). **F** The synergistic score of DOX combined with ISL calculated by using the Synergyfinder 3.0 software. *: *P* < 0.05. **: *P* < 0.01. ***: *P* < 0.001

To demonstrate the synergistic action of ISL and DOX, we firstly verified the inhibitory effect of free-DOX (Fig. 1A) and free-ISL (Fig. 1B) on 4T1 cell, separately. The results showed that both of DOX and ISL revealed a concentration-dependent inhibition of cell viability, and the half maximal inhibitory concentration (IC50) values of DOX (Fig. 1C) and ISL (Fig. 1D) on 4T1 cells were 2.14 µM and 52.58 µM, respectively. Nest, to evaluate the synergistic effects of DOX and ISL, 4T1 cells were treated with DOX alone or in combination with ISL. As descripted in Fig. 1E, the combination of 1.25 µM of DOX and 25 µM of ISL revealed the inhibitory effects of 63.5 ± 0.9%, which was stronger than the arithmetical overlay of respectively 33.9 ± 4.3% for 1.25 µM of DOX and 28.6 ± 2.1% for 25 µM of ISL.

In addition, the Synergyfinder 3.0 software was next used to analyze the synergistic effects of two drugs. As depicted in Fig. 1F, the synthesis score of ISL combination of DOX was 17.56, indicating that DOX combined with ISL revealed synergistic inhibitory effect on 4T1 cells within the studied dose range. Furthermore, the cell viability inhibitory efficacy of 4T1 cells was stronger when DOX and ISL were combined than each drug used separately.

### Preparation and characterization of M(I + D)PH

**Fig. 2:**
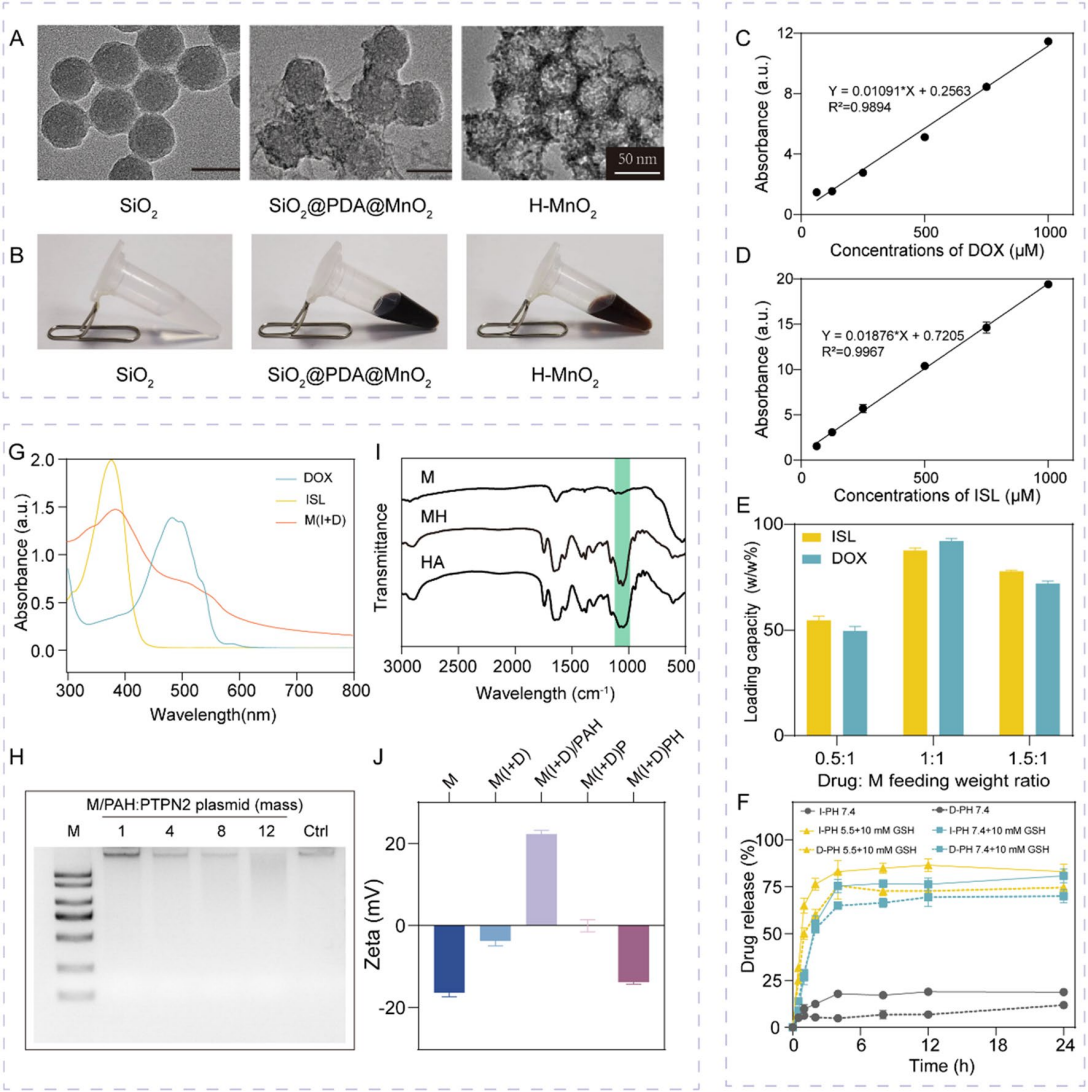
Characterization of M(I + D)PH nanoparticles. **A** TEM images of SiO_2_, SiO_2_@PDA@MnO_2_ and H-MnO_2_ nanoparticles. Scale bar: 50 nm **B** Representative photos of SiO_2_, SiO_2_@PDA@MnO_2_, H-MnO_2_. **C–D** The linear relationship between the UV–vis absorbance and concentration of DOX **C** and ISL **D** (DOX: 485 nm, ISL: 365 nm). **E** The loading weight ratios of drugs in H-MnO_2_ nanoparticles obtained at different feeding drug: H-MnO_2_ ratios. **F** Drugs release profiles from M(I + D) nanoparticles in different conditions within 24 h (n = 3). **G** The UV–vis spectra of DOX, ISL and M(I + D). **H** Agarose gel electrophoresis of M/PAH nanoparticles loading plasmids compared to the control (0.5 µg). **I** FTIR spectra of HA, M and MH nanoparticles. **J** The zeta potential of M, M(I + D), M(I + D)/PAH, M(I + D)P and M(I + D)PH nanoparticles (n = 3)

We synthesized the SiO_2_ nanoparticles following the protocols reported by previous literature. The transmission electron microscope (TEM) images showed that the SiO_2_ (Fig. 2A) nanoparticle was synthesized with a diameter of about 50 nm, and the surface of SiO_2_@PDA@MnO_2_ covered a layer of MnO_2_. Finally, H-MnO_2_ was obtained by etching with sodium hydroxide, and the TEM images showed that H-MnO_2_ had a uniform hollow structure which had a good drug-carrying capacity. M(I + D)P (H-MnO_2_(ISL + DOX)-PTPN2, M(I + D)P) nanoparticles were generated after the ISL, DOX and Cas9/sg-PTPN2 plasmid being loaded, which were further enwrapped with HA to synthesize M(I + D)PH (Fig. S1 and Fig. S2). The representative pictures in the process of synthesis were shown in Fig. 2B.

The hollow structure of H-MnO_2_ with mesoporous shells are expected to be ideal for efficient drug loading. To verify the precise release of the nanoparticles at the tumor site, we examined the drug loading and release capacity of the nanoparticles separately, which was calculated based on the corresponding standard curves (Fig. 2C–D). For drug loading, M was mixed with different concentrations of drugs in dark for 12 h. At the feeding weight ratio (Drug: M) of 1:1, the DOX and ISL loading researched a rather high ratio of 92.03% and 87.53% (Fig. 2E). Then, the drug release behaviors of ISL and DOX from M(I + D) (H-MnO_2_(ISL + DOX), M(I + D)) (Fig. 2F) and M(I + D)PH were then studied in solutions at different solutions. In the presence of 10 mM GSH of PH 7.4, the release of DOX and ISL respectively were 69.96% and 80.72%. Negligible drug release was observed in 24 h in PBS buffer at PH 7.4. Moreover, in the presence of 10 mM GSH of PH 5.5, DOX release quantity reached 74.54% and ISL release quantity reached 83.05%. In addition, the decoration of HA had no obstruction on drug release (Fig. S4). Therefore, the degradation of the nanoparticles was PH/GSH-responsive, which proved that it had good targeting and can reduce the toxic and side effects of the drug on other tissues.

After that, UV–vis spectra were recorded to identify the different drugs and nanoparticles generated during the drug loading (Fig. 2G). Different from the spectrum of H-MnO_2_ (Fig. S3), the emerging absorption peak of M(I + D) nanoparticles at 386 nm was ascribed to the loading of ISL and 480 nm was due to the loading of DOX. Accordingly, the surface charge of these nanoparticles was also measured to monitor the modification process. The M nanoparticle exhibited a strong negative surface. The zeta potentials of products (H-MnO_2_(ISL + DOX)/PAH, M(I + D)/PAH) in the packaging PAH process changed obviously. Then, M(I + D)P nanoparticles were obtained in virtue of PTPN2 CRISPR plasmid loading. The final product M(I + D)PH possessed a negative surface with high negative charged surface (Fig. 2J). The mass ratio of M/PAH (H-MnO_2_/PAH, M/PAH) to PTPN2 plasmid was fixed at 12, which was determined by agarose gel electrophoresis (Fig. 2H). The typical signal at 1152 cm^−1^ was observed in the Fourier transform infrared (FTIR) spectroscopy (Fig. 2I) and agarose gel electrophoresis of M(I + D)PH (Fig. S5), confirming the successful modification of HA. Taken together, we successfully synthesized and characterized the nanoparticles with excellent drug loading and plasmid adsorption capacity, which were also allowed for efficient release.

### Cell viability and metastasis inhibition of M(I + D)PH in vitro

After successful assembly and characterization of M(I + D)PH, the cytotoxicity of MH was evaluated by MTS assay. Over 90.93% cells survived when the concentration of nanoparticles raised to 100 µg/ mL, which showed that the nanocarrier was with a favorable biological safety (Fig. S7). We also verified the cell-killing ability of M(I + D)PH, and there were 38.24% cells survived when the concentration of nanoparticles was 10 µg/mL (Fig. S10).

**Fig. 3:**
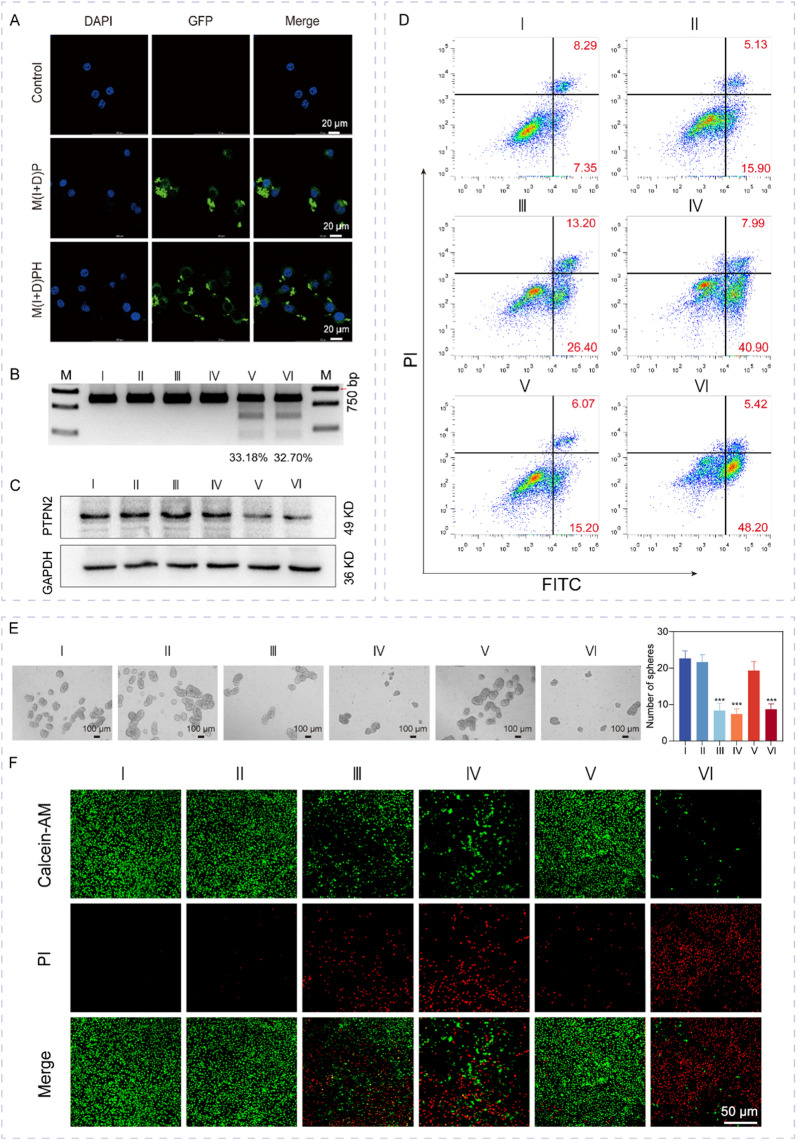
Cell viability and gene editing mediated by M(I + D)PH in vitro. **A** Cellular uptake of M(I + D)P and M(I + D)PH nanoparticles. Scale bar: 20 µm. **B** Evaluation of PTPN2 gene editing efficiency in 4T1 cells with indicated treatments. **C** Protein expression level was detected by western blotting. **D** Representative images for flow cytometry of 4T1 cells with different treatments. I: PBS, II: MH, III: I + D, IV: M(I + D)H, V: MPH, VI: M(I + D)PH. **E** Sphere-forming capacities of 4T1 cells cultured with different treatments. Quantification of sphere number was shown. ***: *P* < 0.001. I: PBS, II: MH, III: I + D, IV: M(I + D)H, V: MPH,VI: M(I + D)PH. **F** The representative fluorescent images of tumor cells by treatment of different treatments dyed with Calcein-AM/PI. Green, Calcein-AM; Red, PI. Scale bar: 50 µm

To verify the intracellular function of the nanoparticles, we tested whether the nanoparticles could enter into the cell successfully. First, 4T1 cells were treated with M(I + D)P and M(I + D)PH nanoparticles for 16 h, respectively. As the designed plasmid encoded the gene of green fluorescent protein (GFP), the fluorescence imaging taken by Confocal Laser Scanning Microscope (CLSM) showed intense green fluorescence signals in the M(I + D)P and M(I + D)PH group compared with the control (Fig. 3A). Flow cytometry was also performed to reveal the successful targeting of nanoparticles to cells. The results demonstrated that the decoration of HA enhanced the targeting of nanoparticles to cancer cells (Fig. S6). These results indicated that nanoparticles were successfully taken up by cancer cells and plasmids were transfected effectively. To further validate the efficiency of genome editing, we cultured 4T1 cell lines with different treatments. T7 Endonuclease I (T7EI) assays were used to assess genome editing efficiency and the groups carried the plasmid showed an evident degree of gene editing, with indels occurring at a frequency of about 33.18% (MPH) and 32.70% (M(I + D)PH) (Fig. 3B and Fig. S8). By contrast, genome editing was not affected in the other groups. We also performed Western Blotting (Fig. 3C and Fig. S9) to confirm that the PTPN2 gene had been successfully knocked down, which demonstrated that our nanosystem had achieved plasmid delivery effects and gene editing efficiency.

Cancer stem cells (CSCs) are a small subpopulation of undifferentiated cells in tumor tissue with strong tumorigenic potential, which were a major cause for the development of drug resistance and tumor relapse. To verify the ability of the drugs to inhibit cancer stem cells, we used sphere-formation assay (Fig. 3E) and colony formation assay (Fig. S12) to verify the inhibitory effect of nanomaterials on cancer stem cells. The results showed that the combination of ISL and DOX effectively reduced spheres and clone formation during cell proliferation. Furthermore, the cell viability inhibition of 4T1 cells after different treatment was determined in vitro. As shown in Fig. 3D and Fig. S11, flow cytometry analysis revealed that M(I + D)PH treatment remarkably increased the apoptosis ratio of 4T1 cells compared with treatment of I + D or M(I + D)H (H-MnO_2_(ISL + DOX)@HA, M(I + D)H) or MPH (H-MnO_2_-PTPN2@HA, MPH). Besides, the results of live/dead fluorescent cell staining (Fig. 3F) detection and the statistical results (Fig. S13A-B) indicated that tumor cells exhibited the highest red intensity (indicated dead cells) and the weakest green intensity (showed live cells) with M(I + D)PH treatment. We also explored adaptability of the synergistic therapy strategy in human breast cancer cells in vitro (Figure S14). The results indicated that our treatment strategy could potentially be applied to clinical practice. In addition, we tested the effect of nanomedicine on DOX-resistant human breast cancer cells. Firstly, we verified the drug resistance of the ADR cells. Notably, WT and ADR cells exhibited different morphology after culture in dish (Figure S15A). The ADR cells had a higher survival rate after 24 h of the anticancer drug DOX treatment (Figure S15B). The inhibitory effect of M(I + D)PH treatment on DOX-resistant cells was evaluated by standard MTS assay (Figure S15C-D). Collectively, these data showed that synthesized M(I + D)PH exhibited robust cancer cell viability inhibition effects with increased cell apoptosis and efficient gene editing in vitro.

### M(I + D)PH improved the in vivo antitumor efficacy

**Fig. 4:**
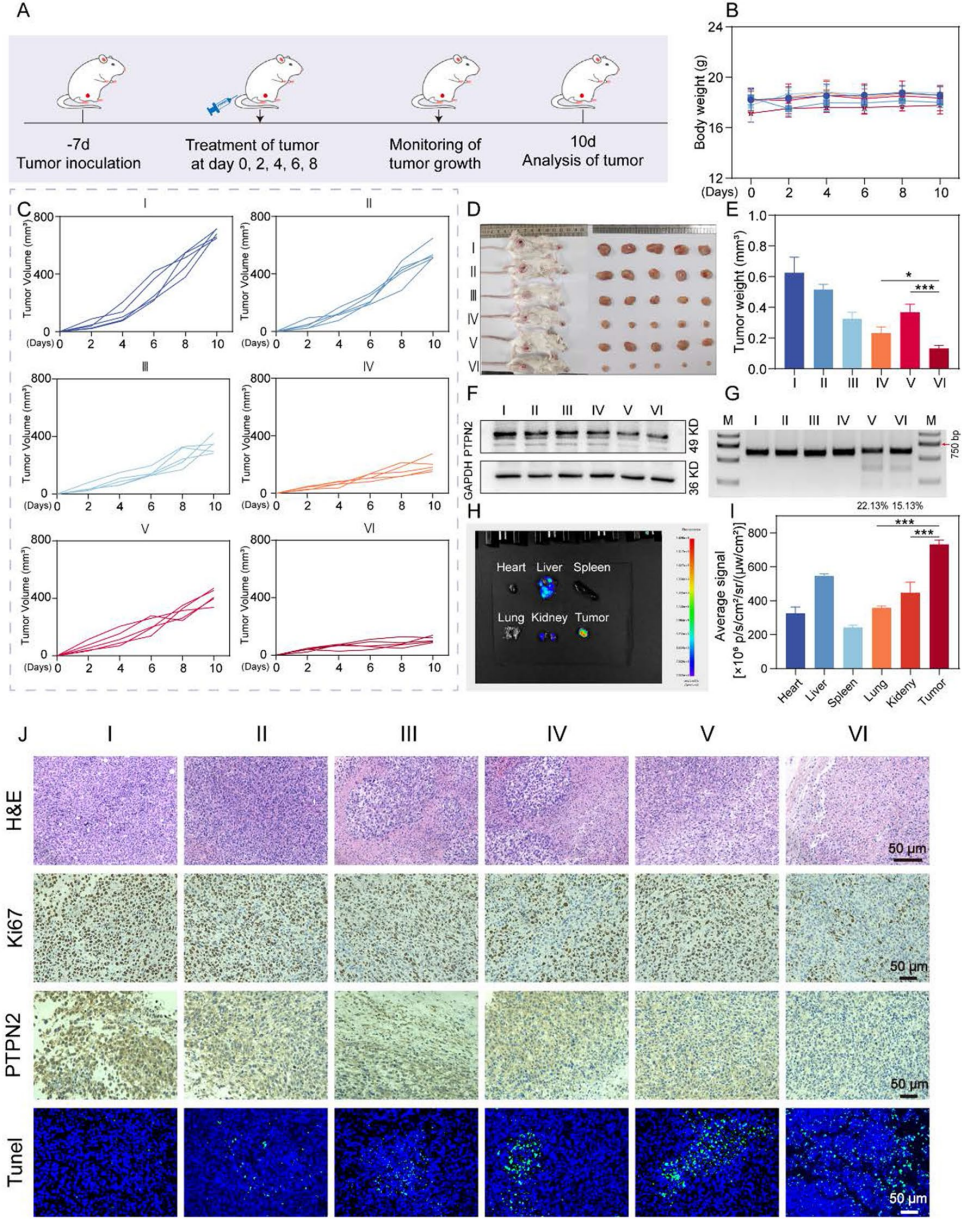
In vivo antitumor efficacy. **A** Schematic illustration of the administration design. **B** Body weight changes of mice after various treatments. **C** Tumor growth curves of 4T1 tumor-bearing mice after intravenous administrations. **D** Representative photos of the sacrificed tumor with different treatments. **E** Tumor weights of the mice. **F** Western blots analysis of PTPN2 and GAPDH expression of the tumor. **G** T7EI assay of tumor tissue after various treatments. **H–I** Representative ex vivo fluorescence image of the major organs and tumor **H** from an 4T1 cells tumor-bearing mouse at 10 h after M(I + D)PH injection and mean fluorescence intensity of major organs and tumor (**I**). **J** Representative images of H&E staining, IHC for Ki67 and PTPN2, and apoptosis in tumor sections. I: PBS, II: MH, III: I + D, IV: M(I + D)H, V: MPH, VI: M(I + D)PH. ***: *P* < 0.001

Inspired by the favorable results in vitro, we continued to explore the antitumor activities of M(I + D)PH in xenograft model, which was developed by subcutaneously injecting 4T1 cells into the right flank regions of mice as shown in Fig. 4A. At the beginning of the experiment, we demonstrated the feasibility of the in vivo experiment by performing hemolysis experiments (Fig. S16). Different concentrations of drugs did not cause significant hemolysis. Similarly, the blood routine tests and blood biochemical tests (Fig. S20) showed that the nanoparticles were safe in vivo. In addition, we performed fluorescence intensity analysis (Fig. 4H–I) about the main organs and tumor tissues of mice, which indicated that the drugs were mainly distributed in the tumor sites at 10 h after nanocarriers injection with active targeting. We also detected the biodistribution of nanoparticles using ICP-MS (Fig. S17). Then, the mice with different treatments were randomly divided into six groups: I (PBS), II (MH), III (I + D), IV (M(I + D)H), V (MPH) and VI (M(=I + D)PH). The mice weight (Fig. 4B and Fig. S23) and tumor volumes (Fig. 4C) of different groups were monitored, and the treatments were performed every two days. The tumor volume of mice in the control group showed a trend of rapid growth, by comparison, the inhibition of tumor growth was significantly stronger in M(I + D)PH group. In the meantime, there was no significant change in body weight during nanodrug administration, which demonstrated the excellent biocompatibility of therapeutic formulation. After 9 days, the mice were sacrificed, and the tumor tissues were harvested and photographed (Fig. 4D). Tumor weight analysis (Fig. 4E) showed the distinct tumor inhibition effect of M(I + D)PH treatment intuitively. Notably, M(I + D)PH treatment showed the greatest inhibitory effect on tumor weight, indicating the synergistic therapeutic efficacy of chemotherapy and the PTPN2 targeted immunotherapy.

The antitumor effect of nanomaterial in vivo was remarkable. Next, we verified its gene-editing efficiency. The T7EI assay (Fig. 4G and Fig. S18) results suggested significant genome disruption in the PTPN2 site in the tumor tissue. The Western Blotting assay (Fig. 4F and Fig. S19) also indicated that the decreased expression of PTPN2. Moreover, the histologic analysis was performed in the tumor sections of mice. As provided in Fig. 4J, the compact violet tumor cells were shown in the control group, but the tumor tissues of M(I + D)PH group exhibited significantly reduced cell density with obvious cell apoptosis and necrosis, which was in consistent with DUTP-biotin-gap terminal labeling (TUNEL) staining. Besides, H&E staining images of the main organs showed no marked pathological damage in treatment groups (Fig. S21). The in-situ Ki-67 staining exhibited a reduction of cell proliferation after treatment with different groups respectively, especially for M(I + D)PH administration. The PTPN2 knockdown efficiency was verified by immunohistochemical staining of the tumor tissues. In summary, our nanoplatform exhibited favorable tumor targeting and significant anti-tumor effect with mild side effects in vivo.

### In vivo antitumor immune response of M(I + D)PH

**Fig. 5:**
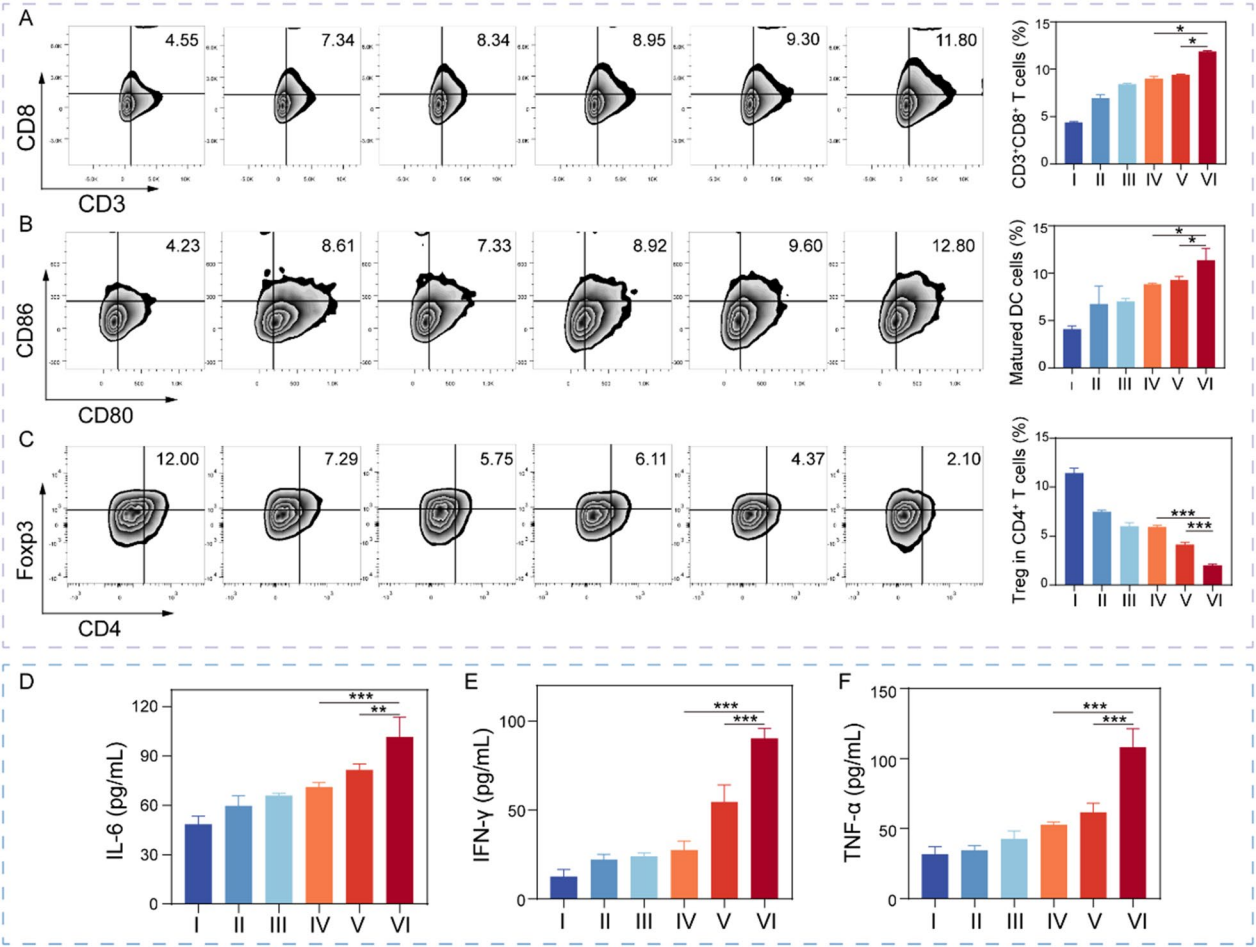
Antitumor immune effects and the related mechanisms. **A–C** Representative flow cytometry plots and the quantitative analysis of CD3^+^ and CD8.^+^T cells (**A**) Matured DCs (**B**) and Treg cells (**C**) in tumor tissues after different treatments. **D–F** The cytokines levels of IL-6 (**D**), IFN-γ (**E**), and TNF-α (**F**) in tumor tissues after different treatments. I: PBS, II: MH, III: I + D, IV: M(I + D)H, V: MPH, VI: M(I+ D)PH. *: *P* < 0.05. **: *P* < 0.01. ***: *P* < 0.001

To clarify the mechanisms of the superior antitumor capability, we collected and analyzed immune cells in the spleen and tumor tissues. Flow cytometry was performed to detect immune cell infiltration. As a critical factor in immunotherapy efficacy, the intratumoral infiltration of cytotoxic T cells, was examined by flow cytometry analysis in the indicated groups. The results demonstrated that the percentage of CD3^+^CD8^+^ T cells was increased in tumor tissues (Fig. 5A) and in spleen (Fig. S22A) with different treatment, notably. The M(I + D)PH group induced the highest level of intratumoral infiltration of CD3^+^CD8^+^ T cells in tumor-bearing mice, which indicated the amplified anti-tumor immunity by DOX/ISL treatment and PTPN2 targeting. Next, we investigated the dendritic cells (DCs) maturation in the tumor sites (Fig. 5B) and spleen (Fig. S22B). The proportion of matured DCs in the M(I + D)PH group (11.3%) was substantially greater than that in the control group (4.1%), proving M(I + D)PH treatment could promote DC maturation in the tumor tissues. Regulatory T cells (Tregs) were a T cell subset with specialized immune functions, which could attenuate the immune response of CTLs and protect tumor tissue from clearance by the immune system. We studied the intratumoral infiltration of Tregs (CD3^+^CD4^+^Foxp3^+^) (Fig. 5C) and the frequency in the spleen (Fig. S22C). The frequency of Tregs in the M(I + D)PH group was much lower compared with the other groups. Taken together, these results implied the successful reprograming of tumor microenvironment with DOX/ISL treatment and PTPN2 targeted immunotherapy.

Many soluble cytokines can be produced by tumor cells as well as tumor-infiltrating immune cells in the TME to regulate the immune responses. Therefore, the production of immune-related cytokines in serum was analyzed by enzyme-linked immunosorbent assay (ELISA). The serum levels of tumor necrosis factor-*α* (TNF-*α*), interleukin-6 (IL-6), and IFN-*γ* in the M(I + D)PH group were significantly elevated compared with the PBS group (Fig. 5D). Taken together, a robust anti-tumor immune response was observed in a reinforced immune microenvironment, suggesting that the M(I + D)PH nanoplatform contributed to the excellent therapeutic effects.

## Conclusion and discussion

In summary, we have successfully constructed a drug delivery system to achieve a combination of traditional chemotherapeutic drugs and promising traditional Chinese medicine, and simultaneously achieving highly efficient gene editing of the cells. In this investigation, the synergistic impact of ISL in conjunction with DOX was validated in breast cancer treatment. HA-encapsulated M(I + D)PH micelles demonstrated obvious cancer stem cell suppression and tumor cell death induction with high gene editing efficiency in vitro. In vivo investigations revealed that M(I + D)PH micelles had active tumor targeting and improved immune activation effects with TME reprogramming. Tumor cytotoxicity was mainly obtained through chemo-immune combo treatment in our synergistic platform.

Drug resistance and the side effects of chemotherapy are challenging issues in clinical treatment. Herbal adjuvant chemotherapy is one of the therapeutic techniques in breast cancer treatment. Chinese herbal ingredient ISL can inhibit cancer cells and increase their sensitivity to chemotherapy. In our investigation, ISL suppressed the development of cancer stem cells and increased the tumor susceptibility to DOX at the cellular level. Significant benefits have also been shown by in vivo therapeutic experiments. As observed, Chinese herbal ISL coupled with DOX could be a powerful candidate for therapeutic treatment of breast cancer.

As a result, DOX and ISL combination therapy with simultaneous delivery carrier may be a viable technique for inhibiting tumor development with enhanced tumor immunity, which may provide a promising strategy for chemotherapeutic resistance therapy. Many bottlenecks encountered by standard chemotherapy treatments are still needed to be resolved, and the anti-cancer impact of traditional Chinese medicine needs to be investigated in the future.

## Materials and methods

### Materials


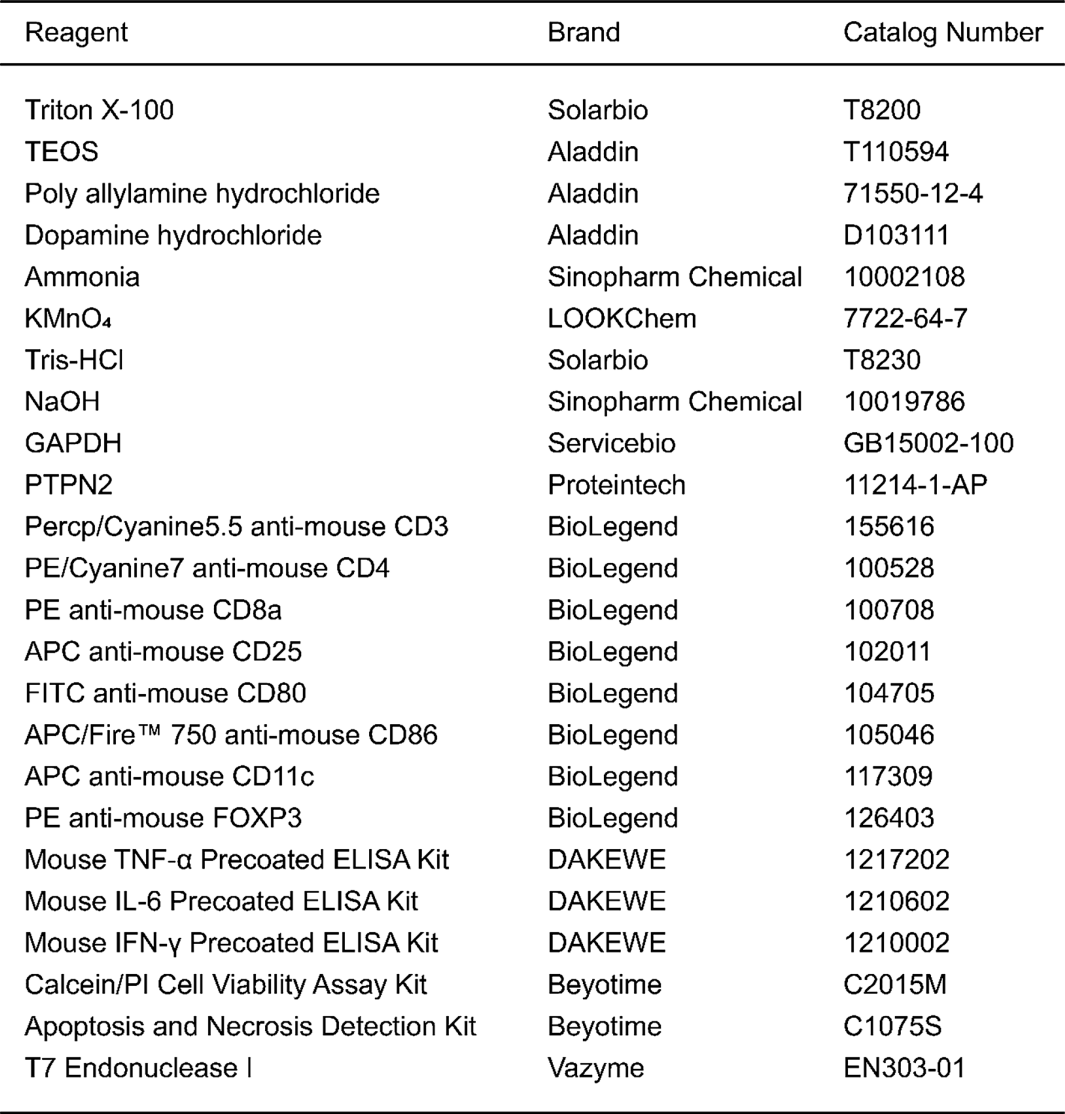


#### Synthesis of H-MnO_2_

The H-MnO2 nanoparticles were synthesized according to the previous literature [37]. Firstly, silica nanoparticles (SiO2) were synthesized as templates. Triton X-100, cyclohexane and n-Hexanol were successively added into conical flask and stirred. Then, a mixture of water and ammonia water was added immediately to the conical flask and continue to stir. Last, TEOS were added, and the mixed solution was stirred for 18 h. Centrifugation was used to collect SiO_2_ nanoparticles. Appropriate proportions of the SiO_2_ and DA were stirred in Tris buffer (10 mM, PH 8.5) at room temperature for about 4 h, also the SiO_2_@PDA was collected by centrifugation. After that, KMnO_4_ aqueous solution was dropwise added into SiO_2_@PDA and kept stirring to wrap a layer of MnO_2_ in its surface. The obtained nanoparticles were collected by centrifugation and etched by NaOH solution (1 M) at 80 ℃ to obtain the H-MnO_2_ nanoparticles.

#### Synthesis of M(I + D)

For ISL and DOX loading, H-MnO2 and different ratio of ISL and DOX in DMSO were stirred overnight under magnetic agitation. Centrifugation was used to extract free drugs, and then washed once with H_2_O obtain M(I + D). The quantity of ISL and DOX was evaluated by respectively monitoring the UV–vis absorption peak at 365 nm and 485 nm after centrifugation.

#### Construction of plasmid

The Cas9/sg-PTPN2 target gene was developed using Feng Zhang's lab's online tool platform (http://crispr.mit.edu/). Plasmid information: pSpCas9(BB)-2A-GFP (PX458) and lentiCRISPR v2 (Ref: Genome engineering using the CRISPR-Cas9 system. Ran FA, Hsu PD, Wright J, Agarwala V, Scott DA, Zhang F. Nat Protoc. 2013 Nov;8(11):2281–308; Improved vectors and genome-wide libraries for CRISPR screening. Sanjana NE, Shalem O, Zhang F. Nat Methods. 2014 Aug;11(8):783–4.).

#### Synthesis of M(I + D)P and M(I + D)PH

For PTPN2 plasmid loading, 1 mL of 10 mg/mL polyacrylate hydrochloric acid (PAH) solution was added to the M(I + D) solution and shaken for 40 min, centrifugation (13000 rpm, 25 min) was used to obtain M(I + D)/PAH nanoparticles and then washed with H_2_O. The PTPN2 plasmid was added into M(I + D)/PAH solution and the mixed solution was shaken for 15 min at 4 °C to obtain M(I + D)P. For HA coating, 1 mg of HA was added into 5 mg of M(I + D)P solution, followed by stirring for 30 min to obtain M(I + D)PH (Table).

#### Drug release study

To investigate ISL and DOX release, a certain amount of M(I + D) was stirred with several buffer solutions (PH 7.4, PH 7.4 + 10 mM GSH, and PH 5.5 + 10 mM GSH) at room temperature. UV–vis spectra were used to determine the quantity of ISL and DOX released at various time points.

#### Cell culture

4T1 cells were grown in DMEM supplemented with 10% FBS and 1% penicillin–streptomycin in a humidified atmosphere of 5% CO_2_/95% air at 37 °C. The multidrug-resistant MCF-7 breast adenocarcinoma cells (MCF-7/ADR) were obtained in BIOESN and cultured following with the protocol.

#### Cell viability assay

4T1 cells (1 × 10^4^ cells/well) were seeded into 96-well plate. Then, the cell was administrated with H-MnO_2_ (0–200 µg/mL) for 24 h. After that, cell viability was measured according to the protocol of MTS. A certain dose of MTS was given, and the culture was continued to avoid light for 1 h, and the results were analyzed at 490 nm with a microplate reader.

#### Western Blotting

Proteins were extracted from cells or tumor tissues and denatured and stored after quantification. The target proteins were separated by 12% sodium dodecyl sulfate polyacrylamide gel electrophoresis (SDS-PAGE). After transmembrane, they were blocked, incubated with primary antibody at 4 ℃ overnight. The other day, it was washed and incubated with secondary antibodies. Finally, signals were observed using TanonTM High-sig ECL Western Blotting substrate.

#### Live-dead cell staining for Calcein/PI

Cells in the log growth phase were seeded in 24-well plates overnight. By different grouping conditions, cells were co-cultured with different administration conditions for 24 h. After that, appropriate volume of Calcein/PI detection working solution was added and incubated at 37 °C without light for 30 min, and the staining effect was observed under a fluorescence microscope.

#### Animal model

All animal procedures were approved and compiled with the guidelines of the Institutional Animal Care and Ethics Committee of Nanjing University of Chinese Medicine (ethical approval number: 202303A049). BALB/c female mice were purchased from Vital River. After adaptive culturing, tumor-bearing mice were constructed by injecting 4T1 cells (1 × 10^6^/100 μl) into the right flank of female mice (5–6 weeks of age). After 7 days’ cell inoculation, tumor volumes reached ~ 60mm^3^, and mice were randomly divided into different groups for further experiments (n = 5 mice/group). The volume of the tumors was recorded every two days. And the tumor volume was calculated as the following formula: volume = 0.5 × (length × width^2^). The mice were sacrificed on the tenth day, we collected the tissue for the subsequent analysis.

#### Flow cytometry analysis

After the tumor tissue was collected, it was crushed and ground. And it was treated with collagenase and Red Blood Cell Lysis Buffer to obtain a single cell suspension. For T cells activation analysis, the cell suspensions were stained with anti-CD3 and anti-CD8 antibodies. For matured DCs activation analysis, the cells were stained with anti-CD11c, anti-CD80 and anti-CD86 antibodies. And for Tregs the cell suspensions were stained with anti-CD4, anti-Foxp3 and anti-CD25. Finally, cells were collected, and analysis was performed with a BD FACSCanto™ II cell analyzer.

#### Histology analysis

The tissues of mice were fixed by immersion in 4% paraformaldehyde. They were then embedded in paraffin and then cut into thin slices and finally stained with H&E. For the immunohistochemistry assays, sections of the tumor tissue samples were dewaxed and hydrated and blocked any endogenous peroxidase activity, after blocking of the nonspecific binding site, sections were incubated with the primary antibodies to Ki67, PTPN2, TUNEL at 4 ℃ overnight. The next day, slides were rinsed and incubated with a horseradish-conjugated secondary antibody (1:1000) for 20 min at 37 °C, and then signal was generated by using the DAB substrate kit.

#### Statistical analysis

All data were obtained from three independent replicate experiments. All the information was presented as means with standard deviations (SD). Data analysis was performed using one-way ANOVA. *P* < 0.05 and *P* < 0.01 were considered to be significant while *P* < 0.001 was considered to be highly significant.

### Supplementary Information

Supplementary Material 1. Additional file 1 of Tumor Microenvironment Responsive Nano-herb and CRISPR Delivery System for Synergistic Chemotherapy and Immunotherapy. Fig. S1. TEM images of M(I+D)PH nanoparticles. Fig. S2. Hydrodynamic size distribution of M(I+D)PH in aqueous suspension measured by the DLS method. Fig. S3. The UV-vis spectra of H-MnO_2_. Fig. S4. Drugs release profiles from M(I+D) PH nanoparticles in different conditions within 24 h (n=3). Fig. S5. Agarose gel electrophoresis of M(I+D)PH compared to M(I+D)P. Fig. S6. 4T1 cells were treated with indicated groups and GFP-positive cells quantified using flow cytometry. Fig. S7. In vitro cytotoxicity of H-MnO_2_@HA nanoparticles at various concentrations in 4T1 cells for 24 h. Fig. S8. The statistical analysis of gene editing efficiency. Fig. S9. The statistical analysis of relative expression of PTPN2. Fig. S10. In vitro cytotoxicity of M(I+D)PH nanoparticles at various concentrations in 4T1 cells for 24 h. Fig. S11. The statistical analysis of apoptosis rate. Fig. S12. Representative photographs of colony formation derived from cells under indicated treatments. Fig. S13. Quantification of Calcein-AM (A). Quantification of PI (B). Fig. S14. Tumor cell viability inhibition and synergistic therapy with M(I+D)H treatment. The representative fluorescent images of cell viability were shown with different treatments in human breast cancer cells. Green, CFSE; Red, PI. Fig. S15. (A) Morphological changes of WT and ADR cells were observed under microscope. (B) Drug resistance to DOX treatment in WT and ADR cells. (C) Morphological changes of ADR cells were observed under indicated treatments. (D) Cell viability of ADR cells was quantified with PBS or M(I+D)PH treatments. Fig. S16. Hemolysis photographs and analysis of red blood cells with different treatments. Fig. S17. Biodistribution of M(I+D)PH via intravenously injection in mice determined using ICP-MS. Fig. S18. The statistical analysis of gene editing efficiency. Fig. S19. The statistical analysis of relative expression of PTPN2. Fig. S20. Blood routine examination and blood biochemistry data of healthy female mice at day 1, 3, and 7 after iv. injection of MH. Fig. S21. H&E staining of major organs and tumor harvested from mice in different groups. Scale bar: 200 µm. Fig. S22. Antitumor immune effects in the spleen. Representative flow cytometry plots and the quantitative analysis of CD3^+^ and CD8^+^ T cells (A) Matured DCs (B) and matured Treg cells (C) in tumor tissues after different treatments. Table: Summary of full name and abbreviation.

## Data Availability

No datasets were generated or analysed during the current study.
