# Supplementary material for: Tumor microenvironment responsive nano-herb and CRISPR delivery system for synergistic chemotherapy and immunotherapy
Source: J Nanobiotechnology. 2024 Jun 19;22:346. doi: 10.1186/s12951-024-02571-9 (PMC11186293; doi:10.1186/s12951-024-02571-9)
Supplement: Supplementary file 1 — Supplementary Material 1. Additional file 1 of Tumor Microenvironment Responsive Nano-herb and CRISPR Delivery System for Synergistic Chemotherapy and Immunotherapy. Fig. S1. TEM images of M(I+D)PH nanoparticles. Fig. S2. Hydrodynamic size distribution of M(I+D)PH in aqueous suspension measured by the DLS method. Fig. S3. The UV-vis spectra of H-MnO2. Fig. S4. Drugs release profiles from M(I+D) PH nanoparticles in different conditions within 24 h (n=3). Fig. S5. Agarose gel electrophoresis of M(I+D)PH compared to M(I+D)P. Fig. S6. 4T1 cells were treated with indicated groups and GFP-positive cells quantified using flow cytometry. Fig. S7. In vitro cytotoxicity of H-MnO2@HA nanoparticles at various concentrations in 4T1 cells for 24 h. Fig. S8. The statistical analysis of gene editing efficiency. Fig. S9. The statistical analysis of relative expression of PTPN2. Fig. S10. In vitro cytotoxicity of M(I+D)PH nanoparticles at various concentrations in 4T1 cells for 24 h. Fig. S11. The statistical analysis of apoptosis rate. Fig. S12. Representative photographs of colony formation derived from cells under indicated treatments. Fig. S13. Quantification of Calcein-AM (A). Quantification of PI (B). Fig. S14. Tumor cell viability inhibition and synergistic therapy with M(I+D)H treatment. The representative fluorescent images of cell viability were shown with different treatments in human breast cancer cells. Green, CFSE; Red, PI. Fig. S15. (A) Morphological changes of WT and ADR cells were observed under microscope. (B) Drug resistance to DOX treatment in WT and ADR cells. (C) Morphological changes of ADR cells were observed under indicated treatments. (D) Cell viability of ADR cells was quantified with PBS or M(I+D)PH treatments. Fig. S16. Hemolysis photographs and analysis of red blood cells with different treatments. Fig. S17. Biodistribution of M(I+D)PH via intravenously injection in mice determined using ICP-MS. Fig. S18. The statistical analysis of gene editing efficien [file 12951_2024_2571_MOESM1_ESM.docx]

**Supporting Information**

**Tumor Microenvironment Responsive Nano-herb and CRISPR Delivery System for Synergistic Chemotherapy and Immunotherapy**

*Yuanyuan Jia*^[a]#^*,* *Yuhui Yao*^[b]#^*,* *Lingyao Fan*^[b]^*, Qiqing Huang*^[a]^*, Guohao Wei*^[b]^*, Peiliang Shen*^[a]^*, Jia Sun*^[a]^*,* *Gaoshuang Zhu*^[a]^*, Zhaorui Sun*^[c]^**, Chuandong Zhu*^[b]^**, Xin Han*^[a]^*

[a] The Second Affiliated Hospital of Nanjing University of Chinese Medicine, Jiangsu Collaborative Innovation Center of Chinese Medicinal Resources Industrialization, School of Medicine, Nanjing University of Chinese Medicine, Nanjing 210023, China

Email: xhan0220@njucm.edu.cn (Xin Han)

[b] Department of Oncology, The Second Hospital of Nanjing, Nanjing University of Chinese Medicine, Nanjing 210003, China

Email: zhucd@njucm.edu.cn (Chuandong Zhu)

[c] Department of Emergency Medicine, Jinling Clinical Medical College, Nanjing University of Chinese Medicine, Nanjing 210002, China

Email: sunzhr84@163.com (Zhaorui Sun)

# These authors contribute equally to this work.


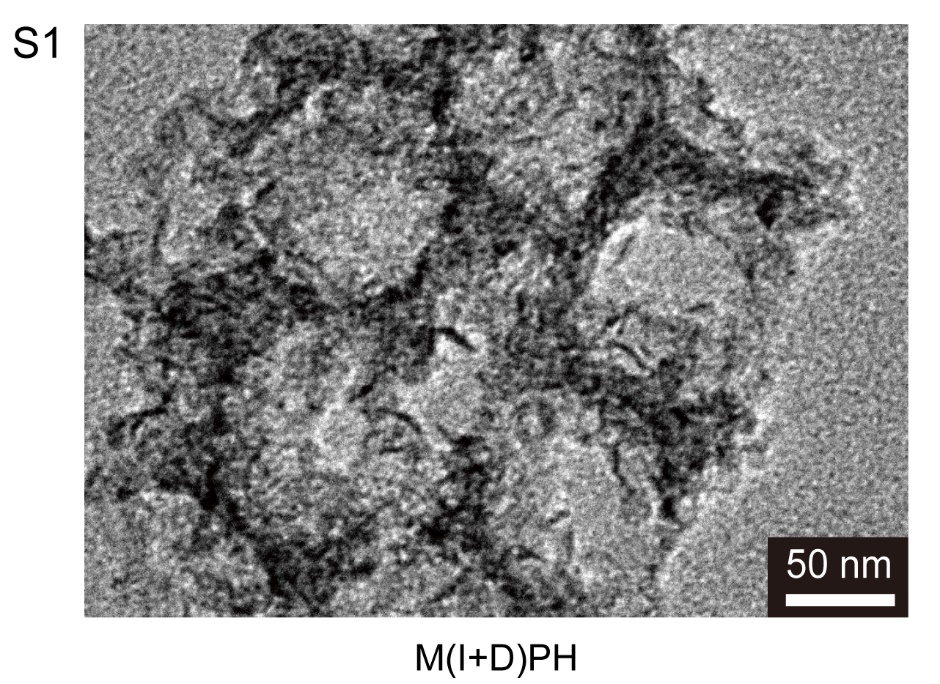


**Fig. S1.** TEM images of M(I+D)PH nanoparticles.


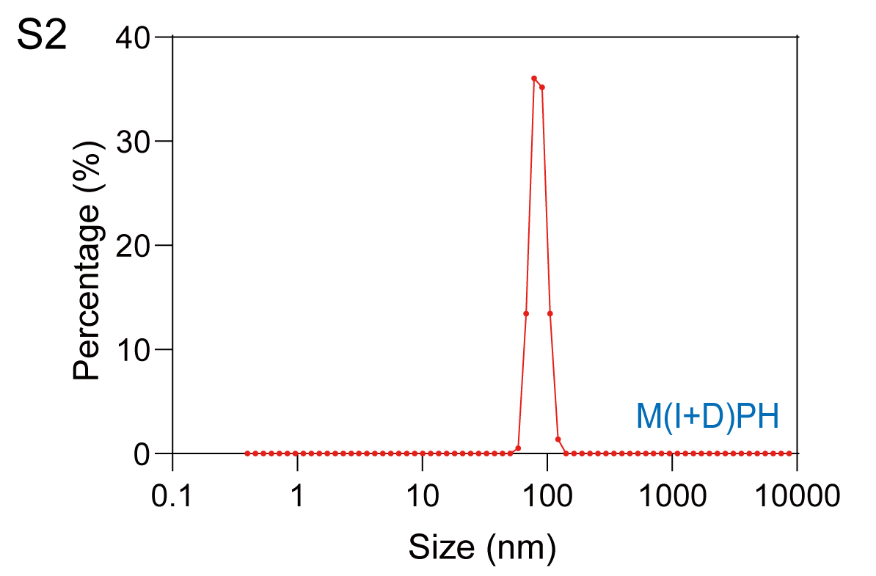


**Fig. S2.** Hydrodynamic size distribution of M(I+D)PH in aqueous suspension measured by the DLS method.


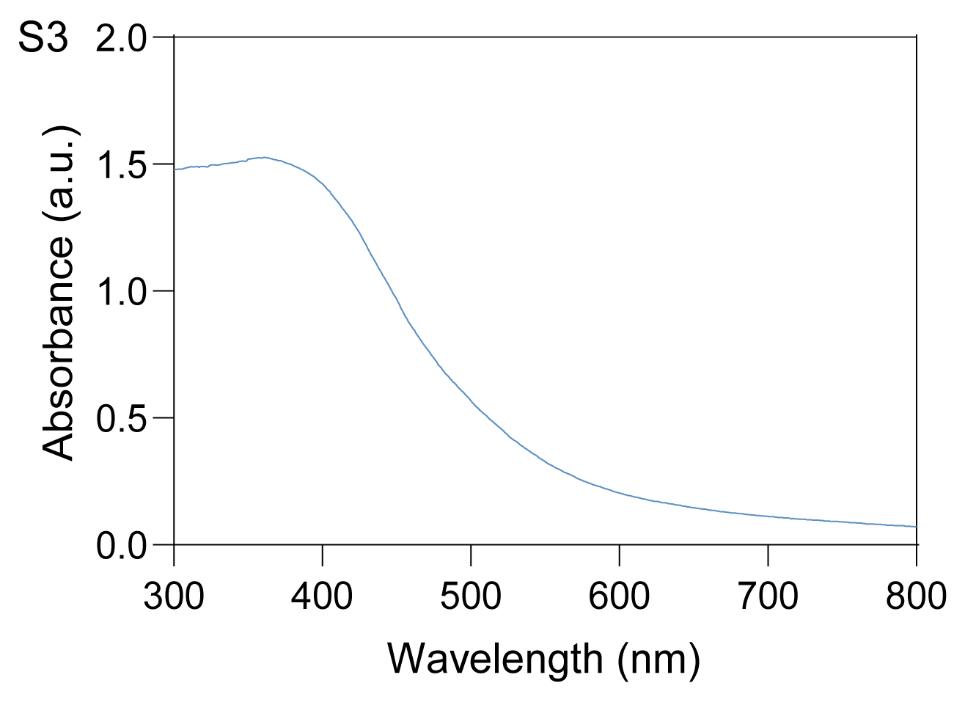


**Fig. S3.** The UV-vis spectra of H-MnO_2_.


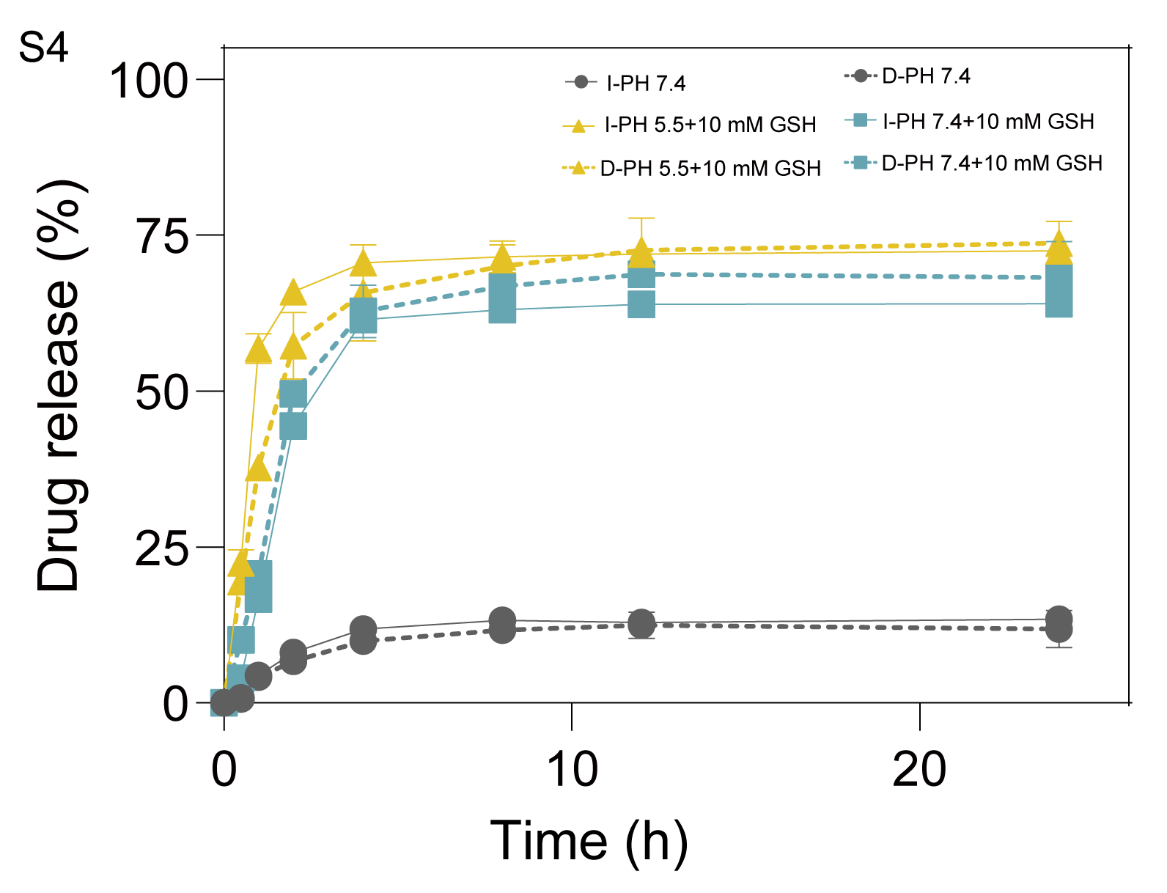


**Fig. S4.** Drugs release profiles from M(I+D)PH nanoparticles in different conditions within 24 h (n=3).


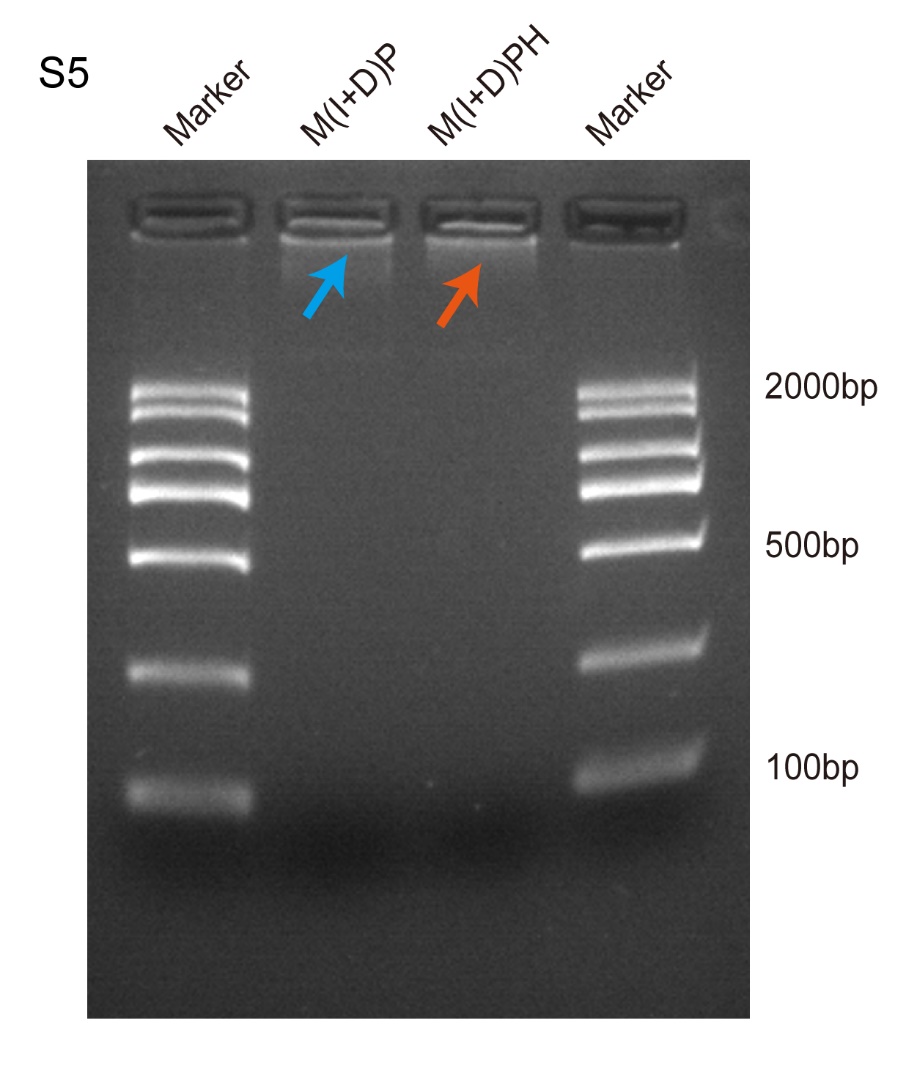


**Fig. S5.** Agarose gel electrophoresis of M(I+D)PH compared to M(I+D)P.


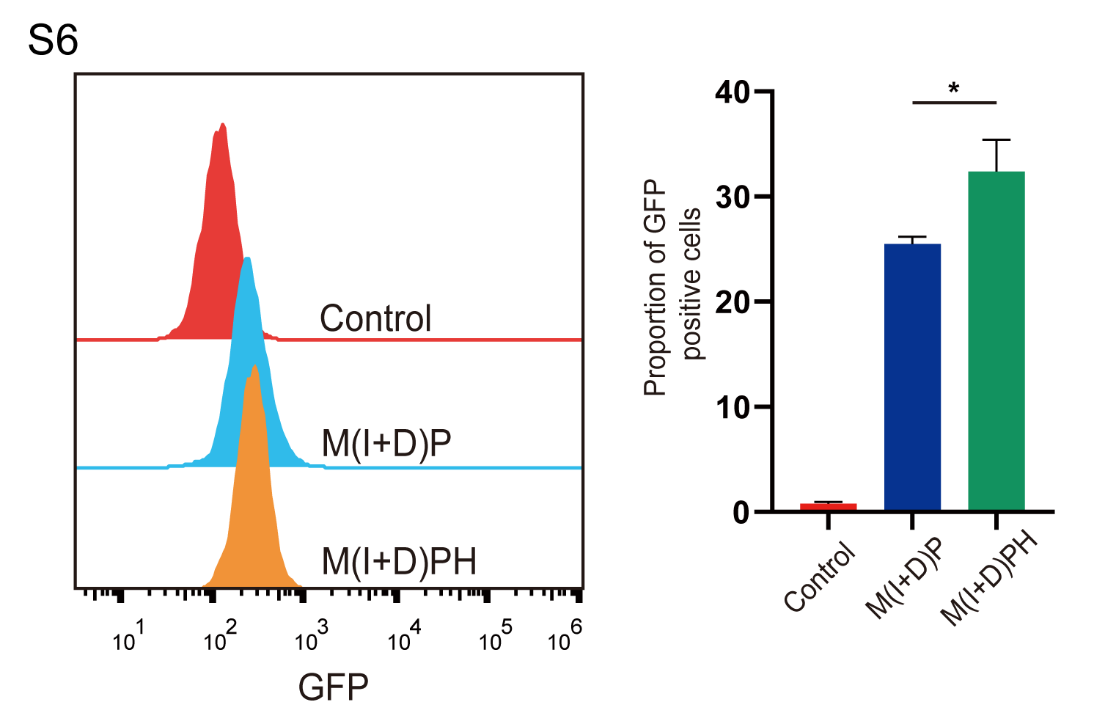


**Fig. S6.** 4T1 cells were treated with indicated groups and GFP-positive cells quantified using flow cytometry.


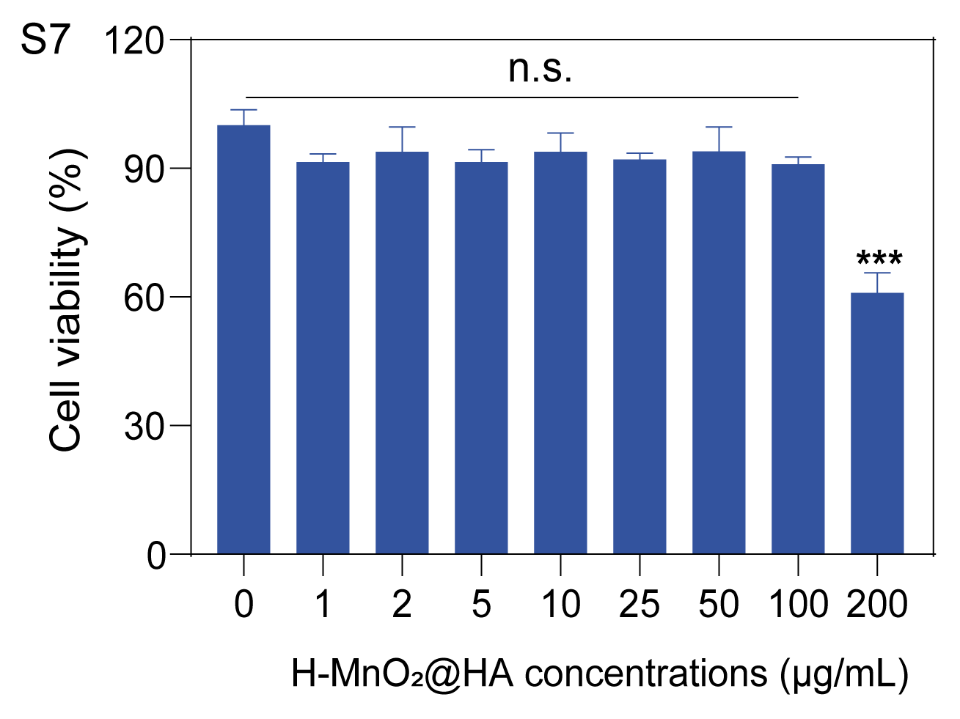


**Fig. S7.** In vitro cytotoxicity of H-MnO_2_@HA nanoparticles at various concentrations in 4T1 cells for 24 h.


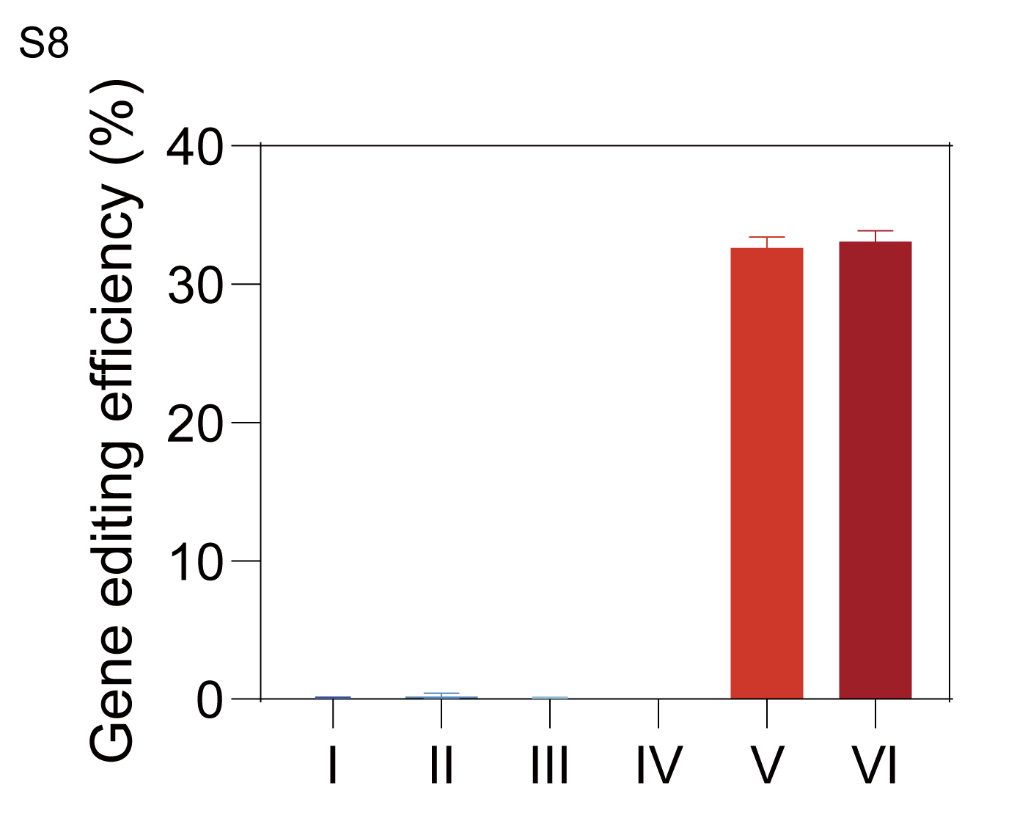


**Fig. S8.** The statistical analysis of gene editing efficiency.


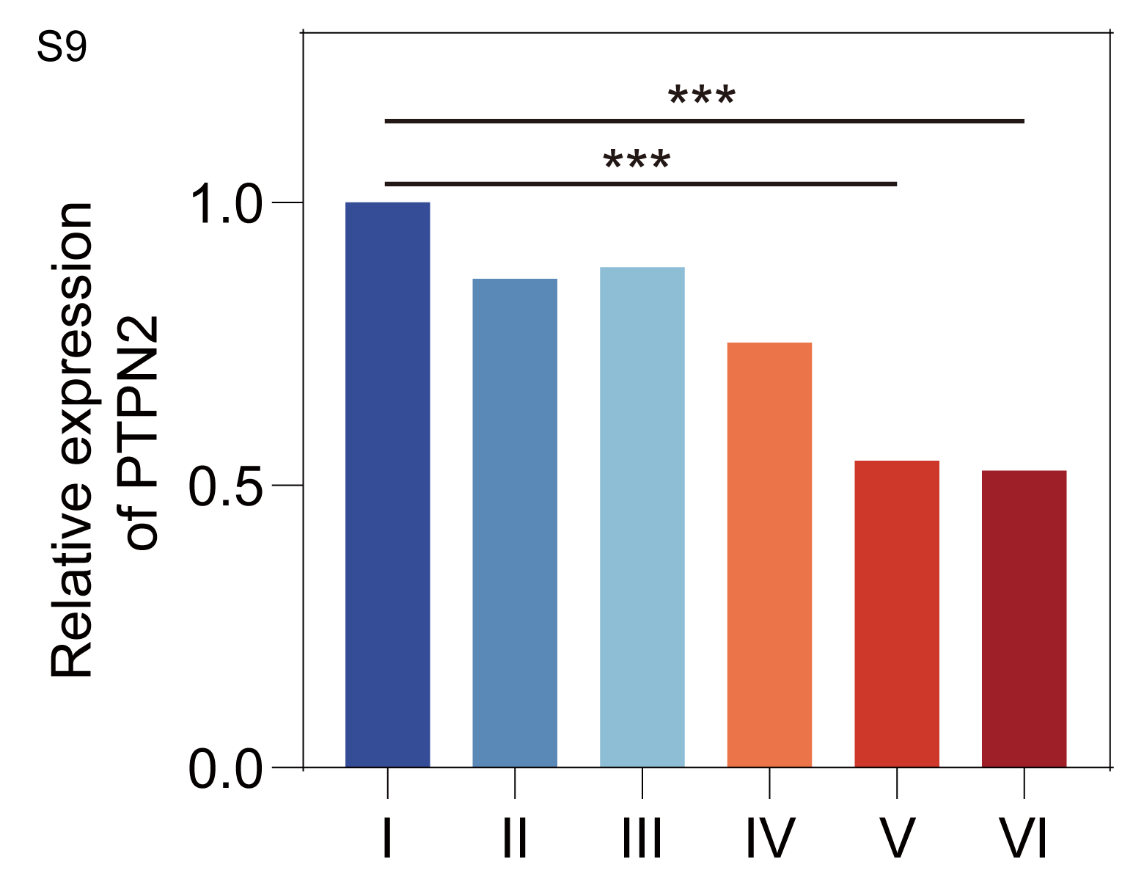


**Fig. S9.** The statistical analysis of relative expression of PTPN2.


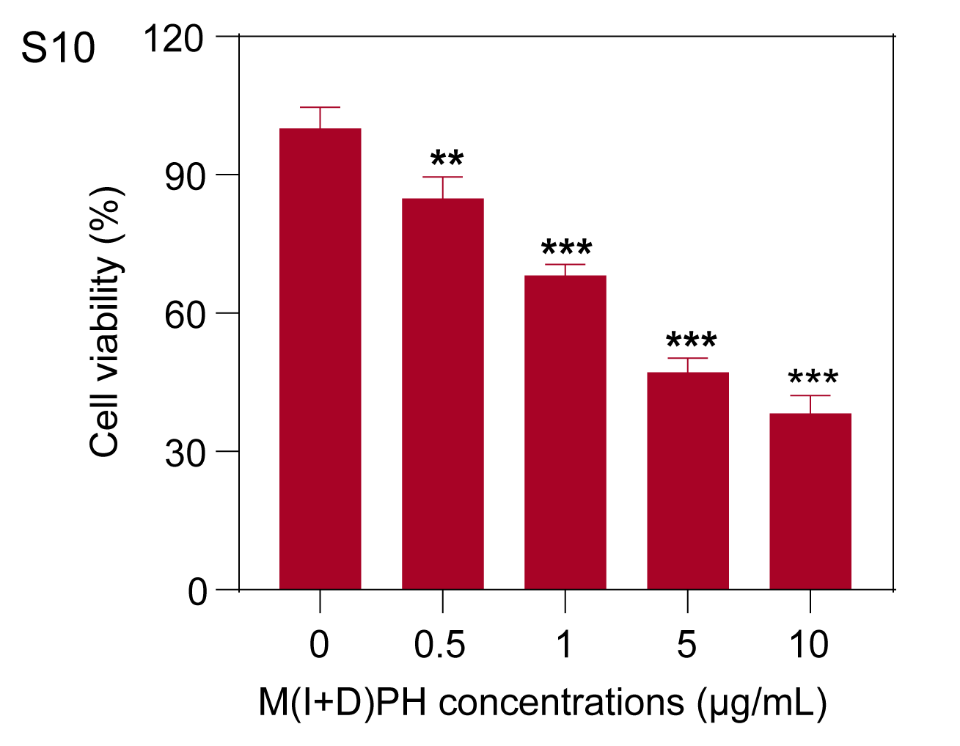


**Fig. S10.** In vitro cytotoxicity of M(I+D)PH nanoparticles at various concentrations in 4T1 cells for 24 h.


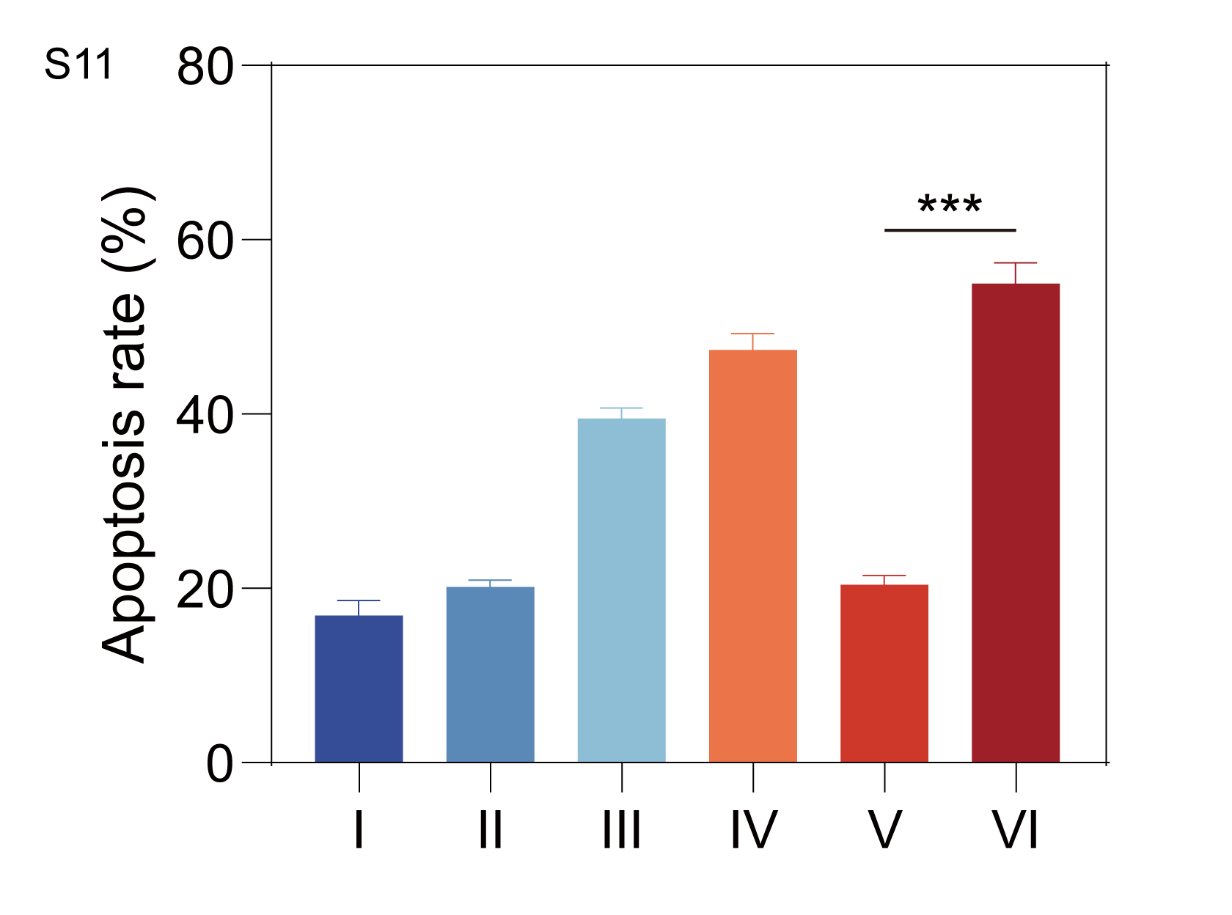


**Fig. S11.** The statistical analysis of apoptosis rate.


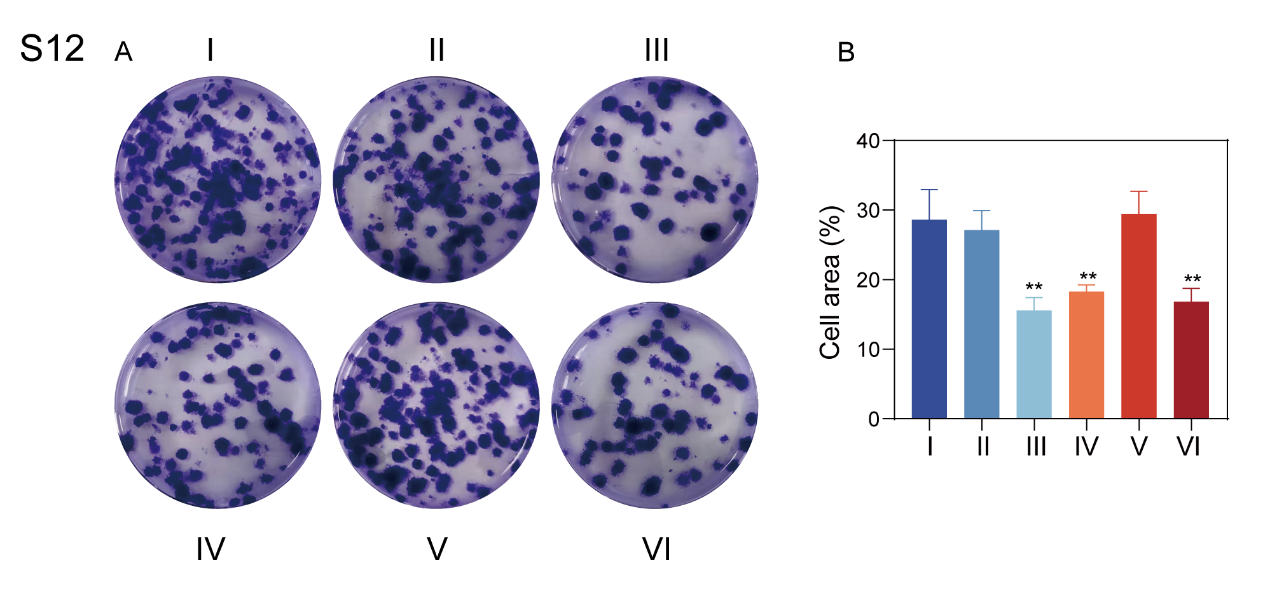


**Fig. S12.** Representative photographs of colony formation derived from cells under indicated treatments.


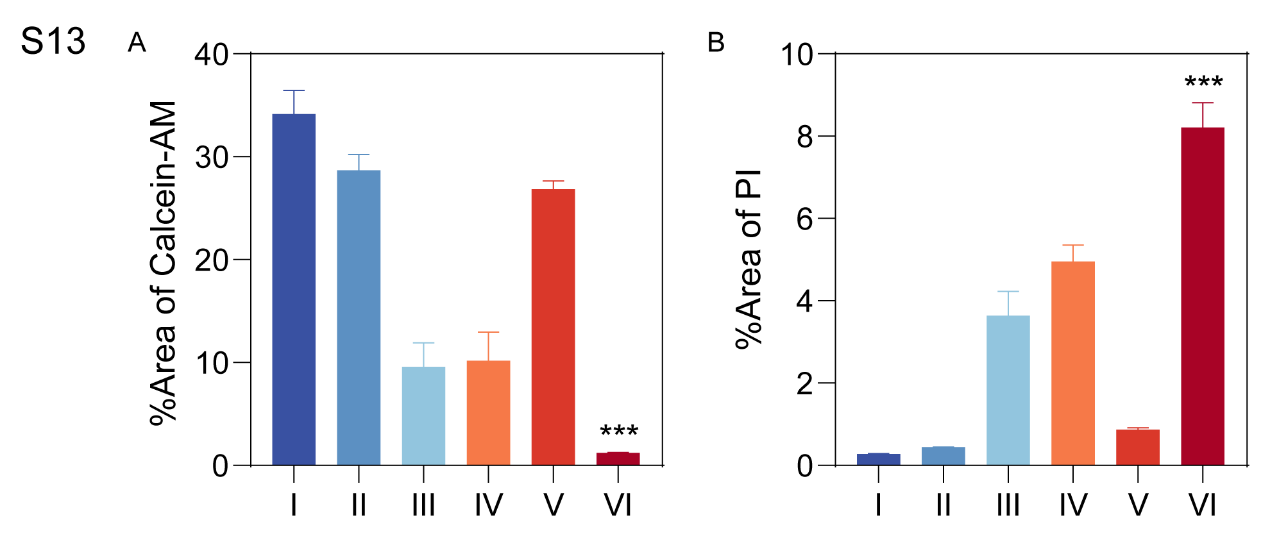


**Fig. S13.** Quantification of Calcein-AM (A). Quantification of PI (B).


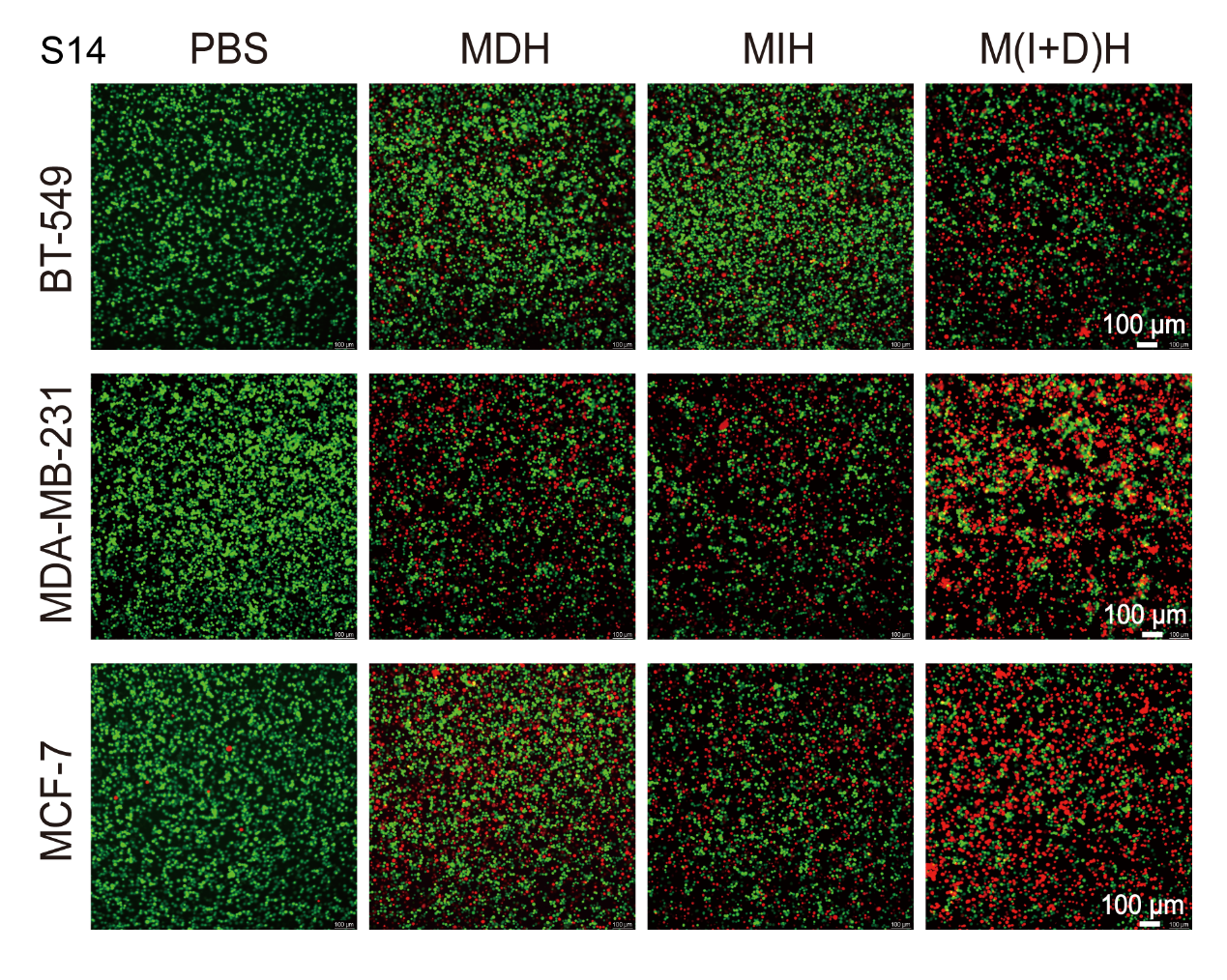


**Fig. S14.** Tumor cell viability inhibition and synergistic therapy with M(I+D)H treatment. The representative fluorescent images of cell viability were shown with different treatments in human breast cancer cells. Green, CFSE; Red, PI.


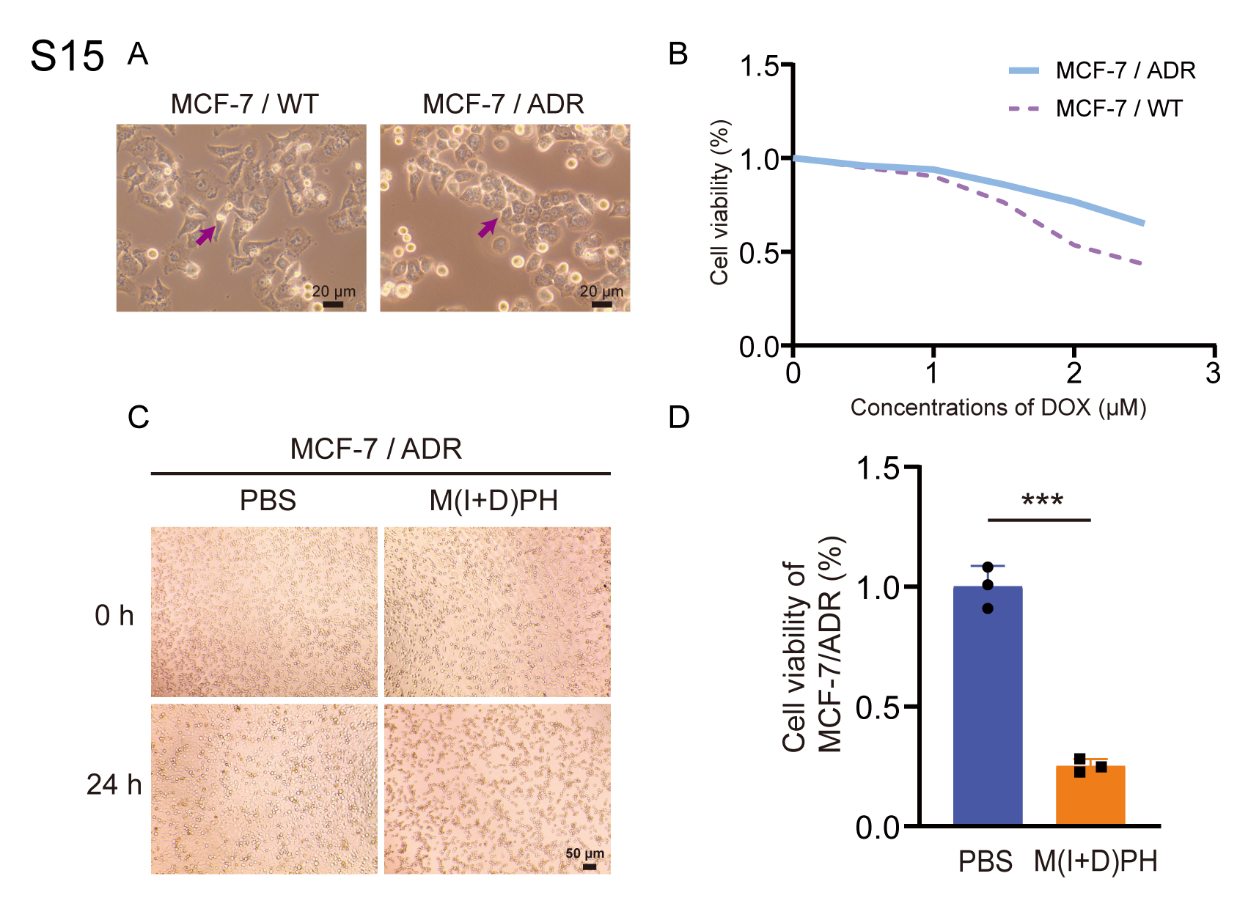


**Fig. S15.** (A) Morphological changes of WT and ADR cells were observed under microscope. (B) Drug resistance to DOX treatment in WT and ADR cells. (C) Morphological changes of ADR cells were observed under indicated treatments. (D) Cell viability of ADR cells was quantified with PBS or M(I+D)PH treatments.


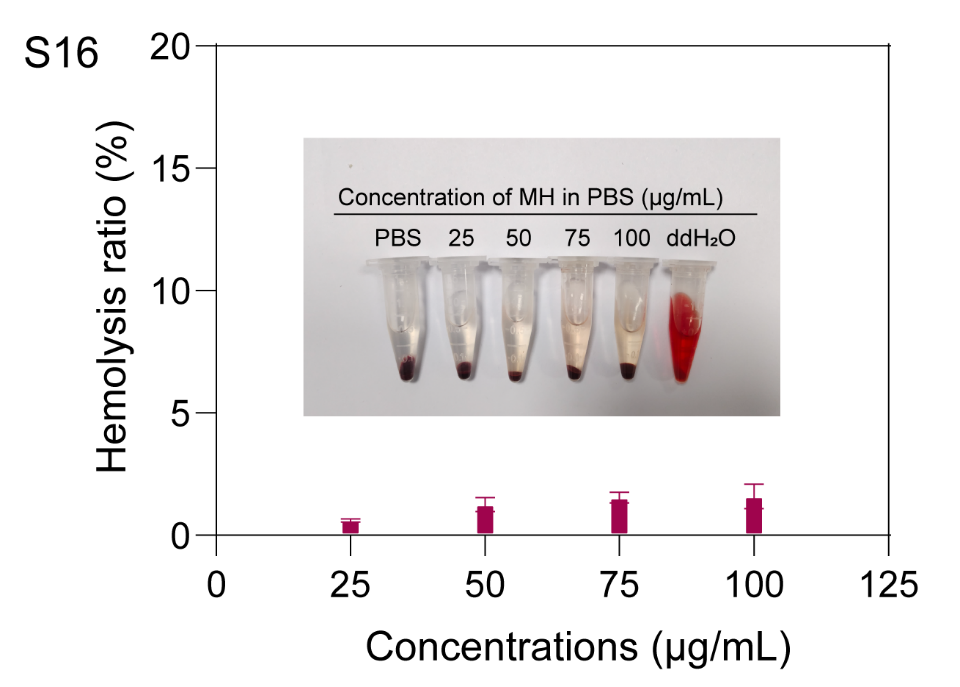


**Fig. S16.** Hemolysis photographs and analysis of red blood cells with different treatments.


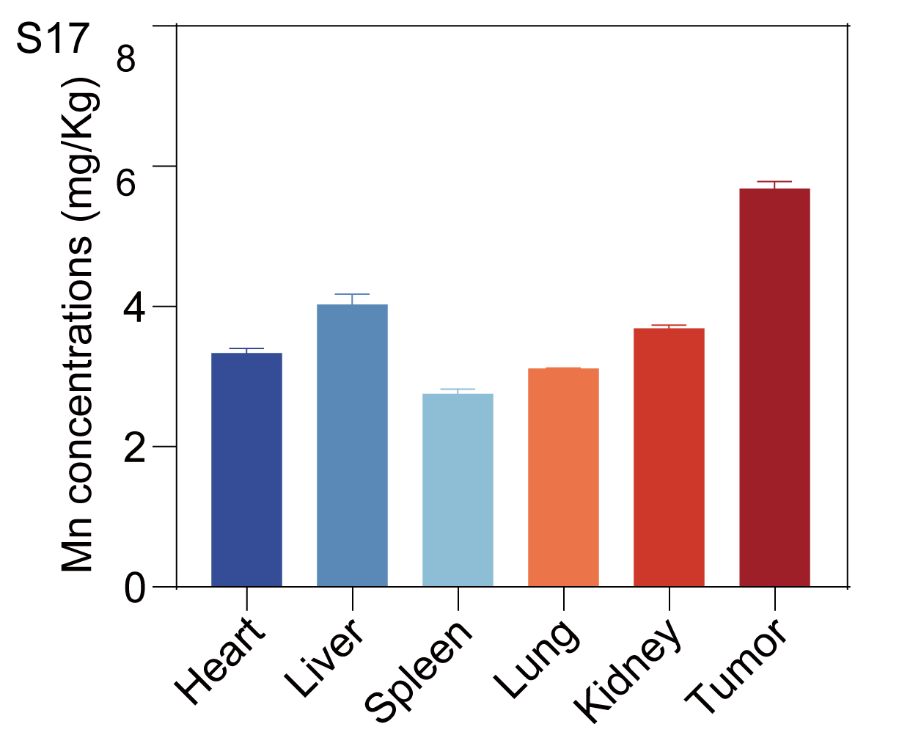


**Fig. S17.** Biodistribution of M(I+D)PH via intravenously injection in mice determined using ICP-MS.


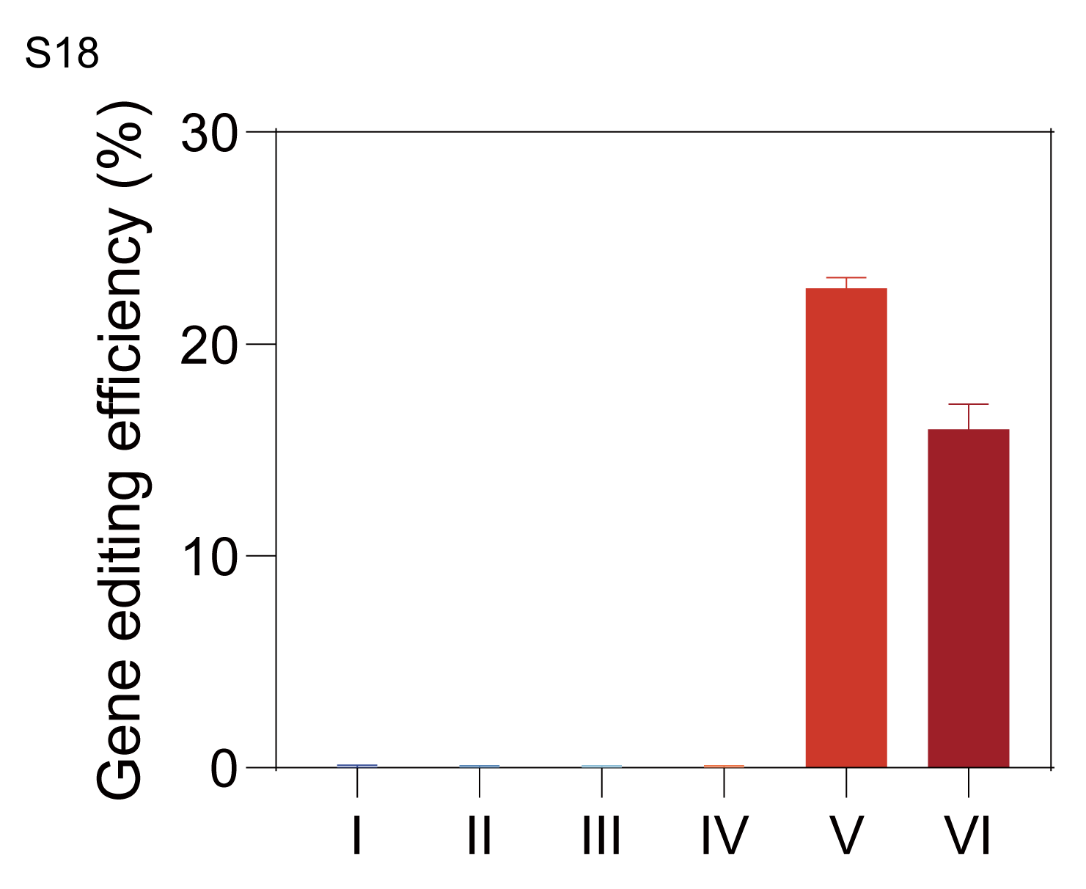


**Fig. S18.** The statistical analysis of gene editing efficiency.


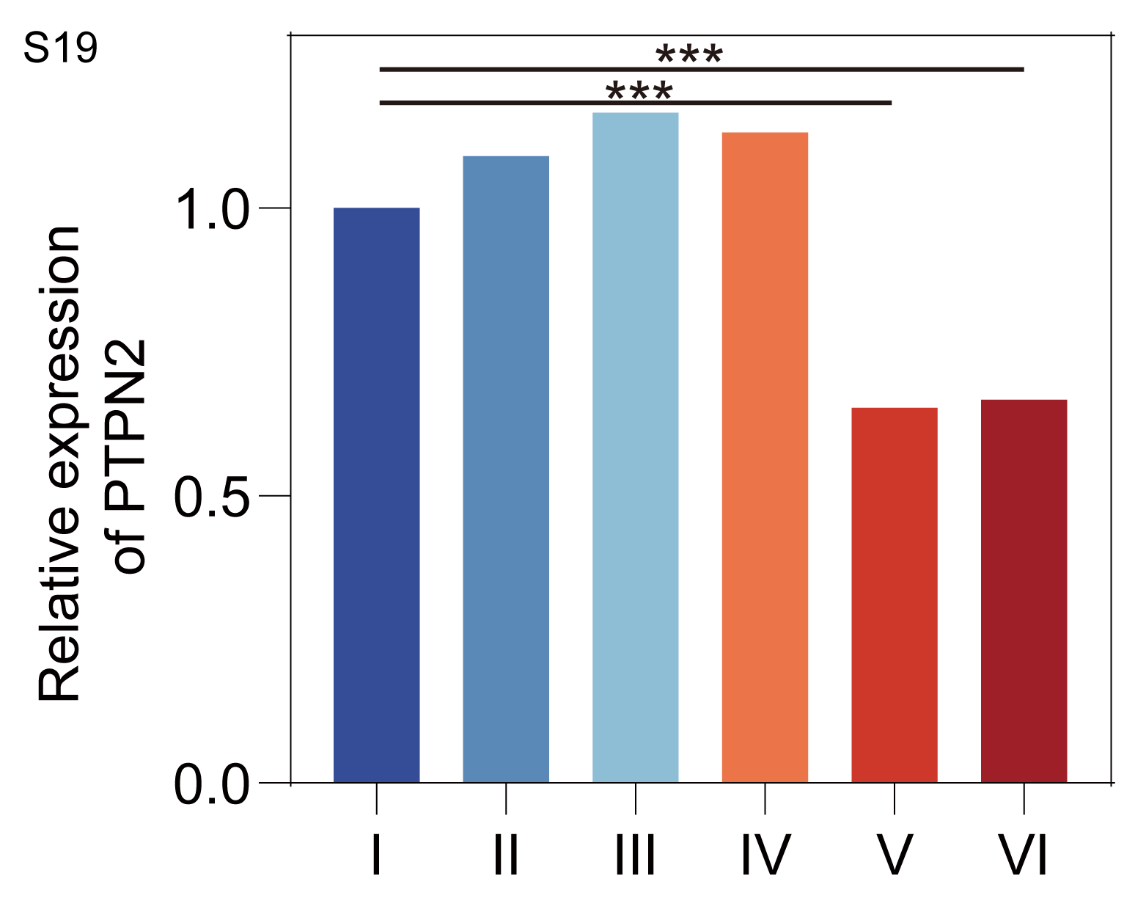


**Fig. S19.** The statistical analysis of relative expression of PTPN2.


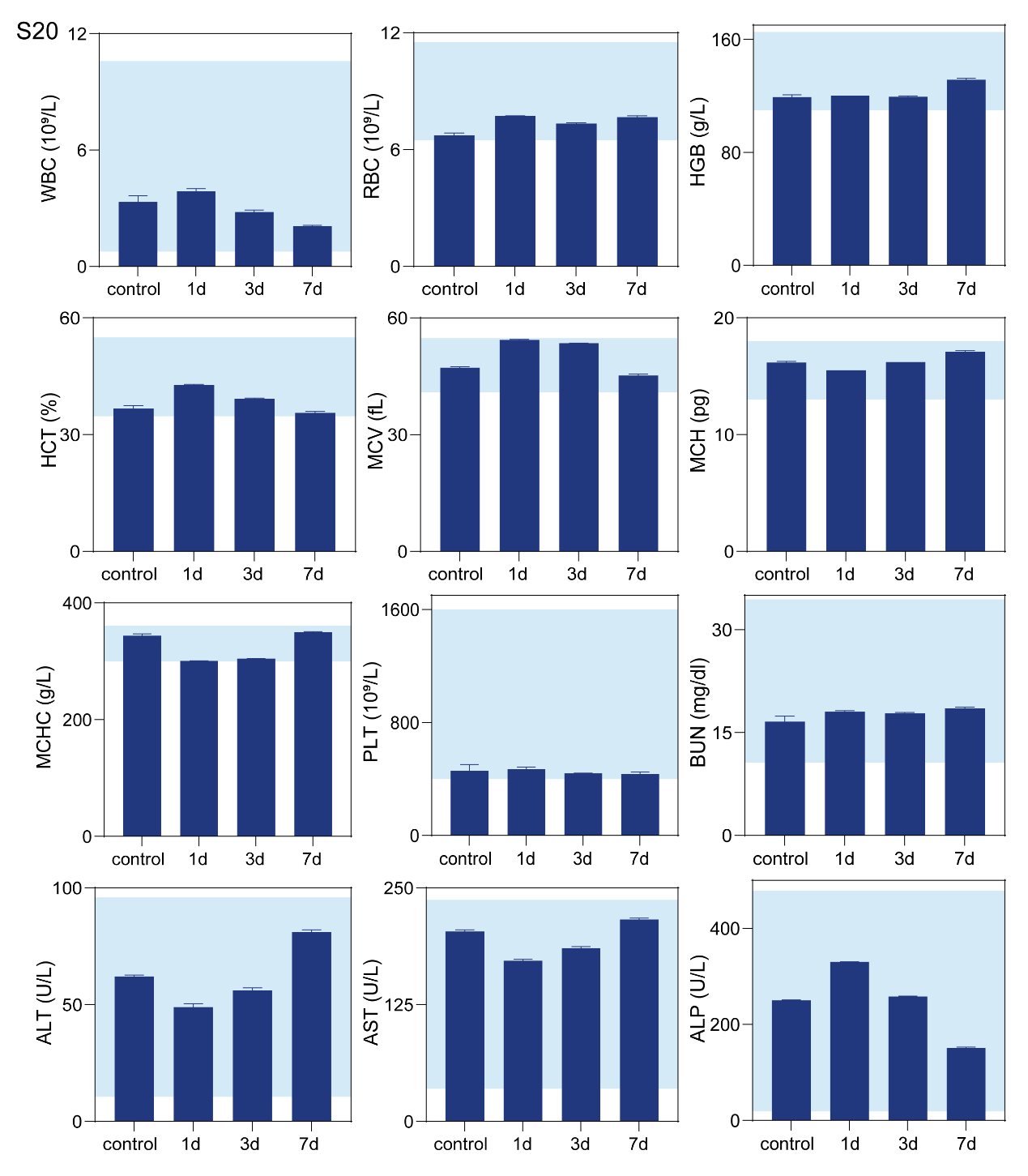


**Fig. S20.** Blood routine examination and blood biochemistry data of healthy female mice at day 1, 3, and 7 after iv. injection of MH. The control group was identified as healthy female mice without any treatment and reference ranges of hematology data of healthy female mice were marked in bule. The examined parameters included white blood cell (WBC) counts, red blood cell (RBC) counts, hemoglobin (HGB), hematocrit (HCT), mean corpuscular hemoglobin concentration (MCHC), mean corpuscular volume (MCV), mean corpuscular hemoglobin (MCH) and platelets (PLT), alanine aminotransferase (ALT), Alkaline Phosphatase (ALP) and Aspartate aminotransferase (AST), and blood urea nitrogen (BUN), (n = 3).


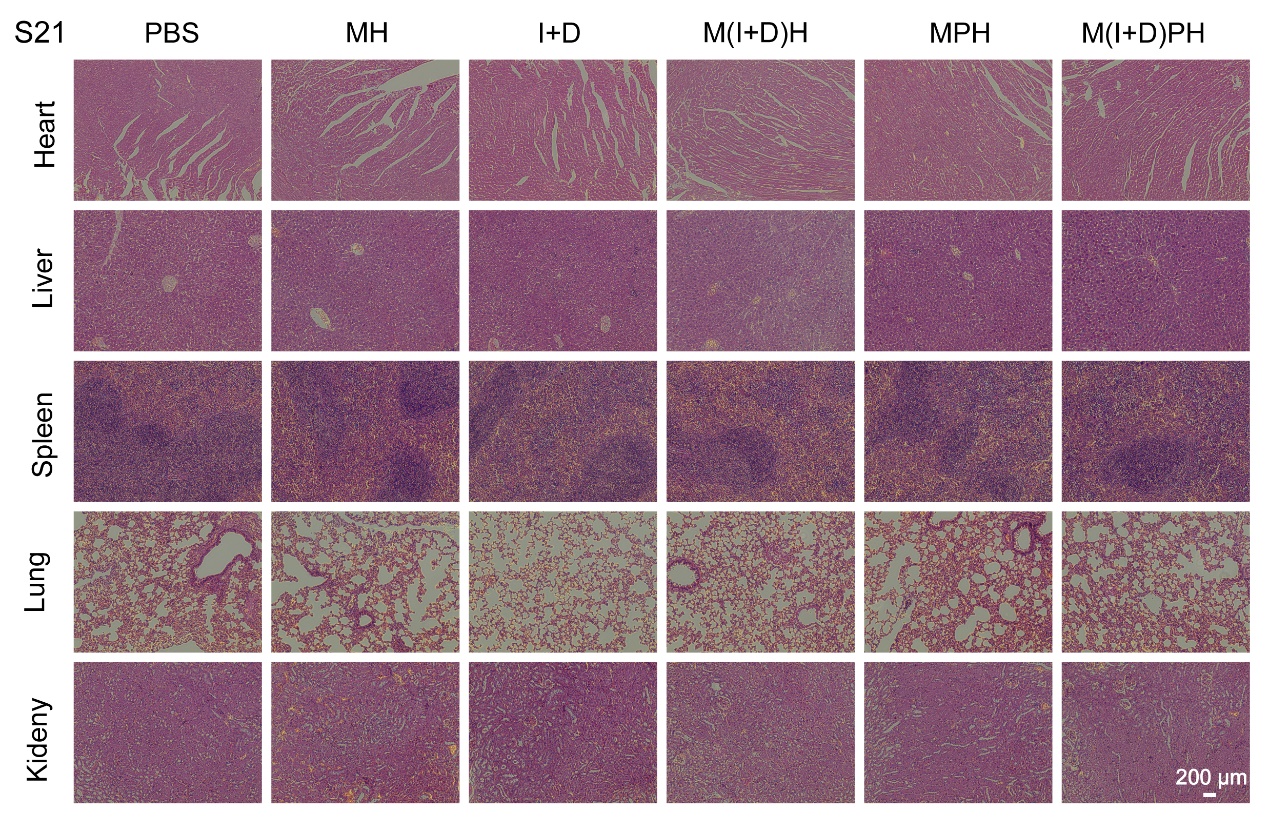


**Fig. S21.** H&E staining of major organs and tumor harvested from mice in different groups. Scale bar: 200 µm.


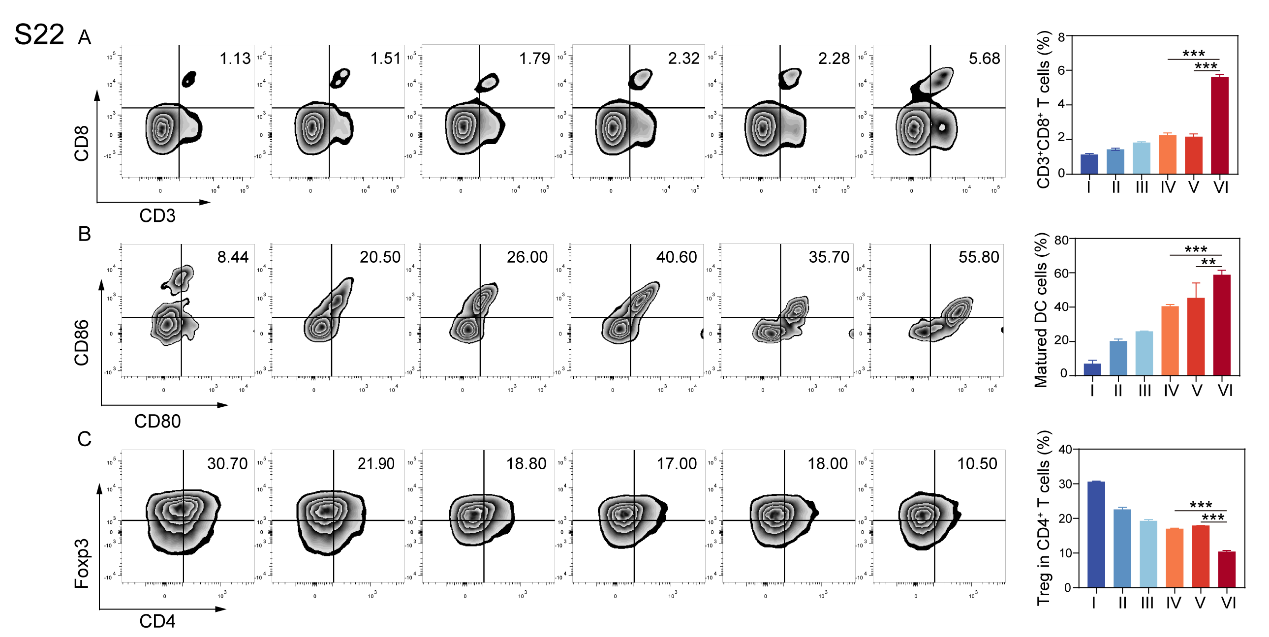


**Fig. S22.** Antitumor immune effects in the spleen. Representative flow cytometry plots and the quantitative analysis of CD3^+^ and CD8^+^ T cells (A) Matured DCs (B) and matured Treg cells (C) in tumor tissues after different treatments.


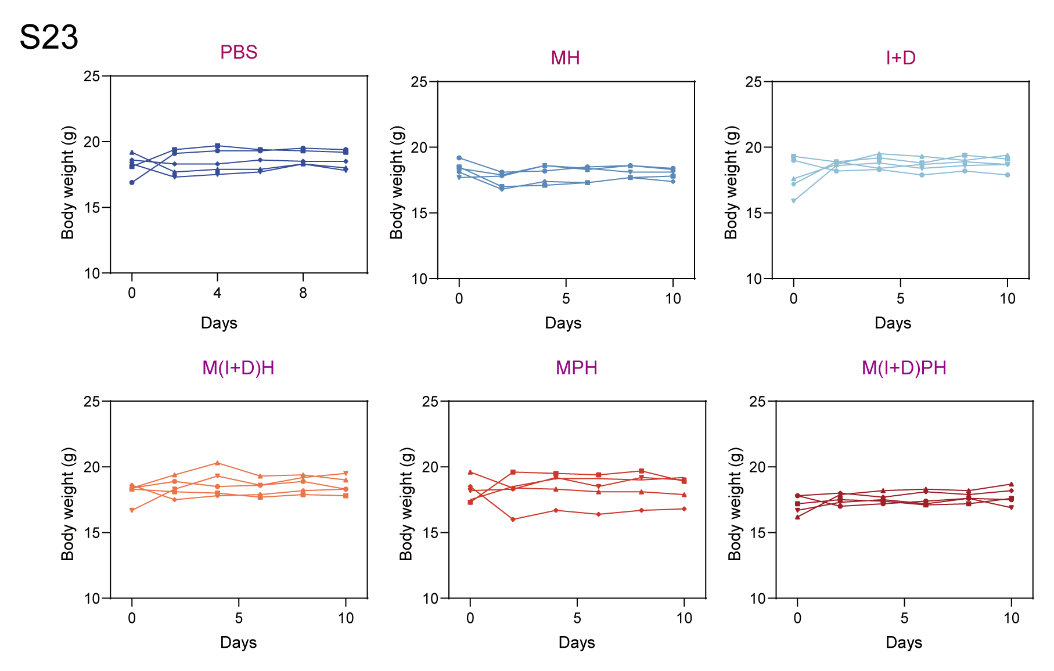


**Fig. S23.** Body weight curves of 4T1 tumor-bearing mice with different treatments.


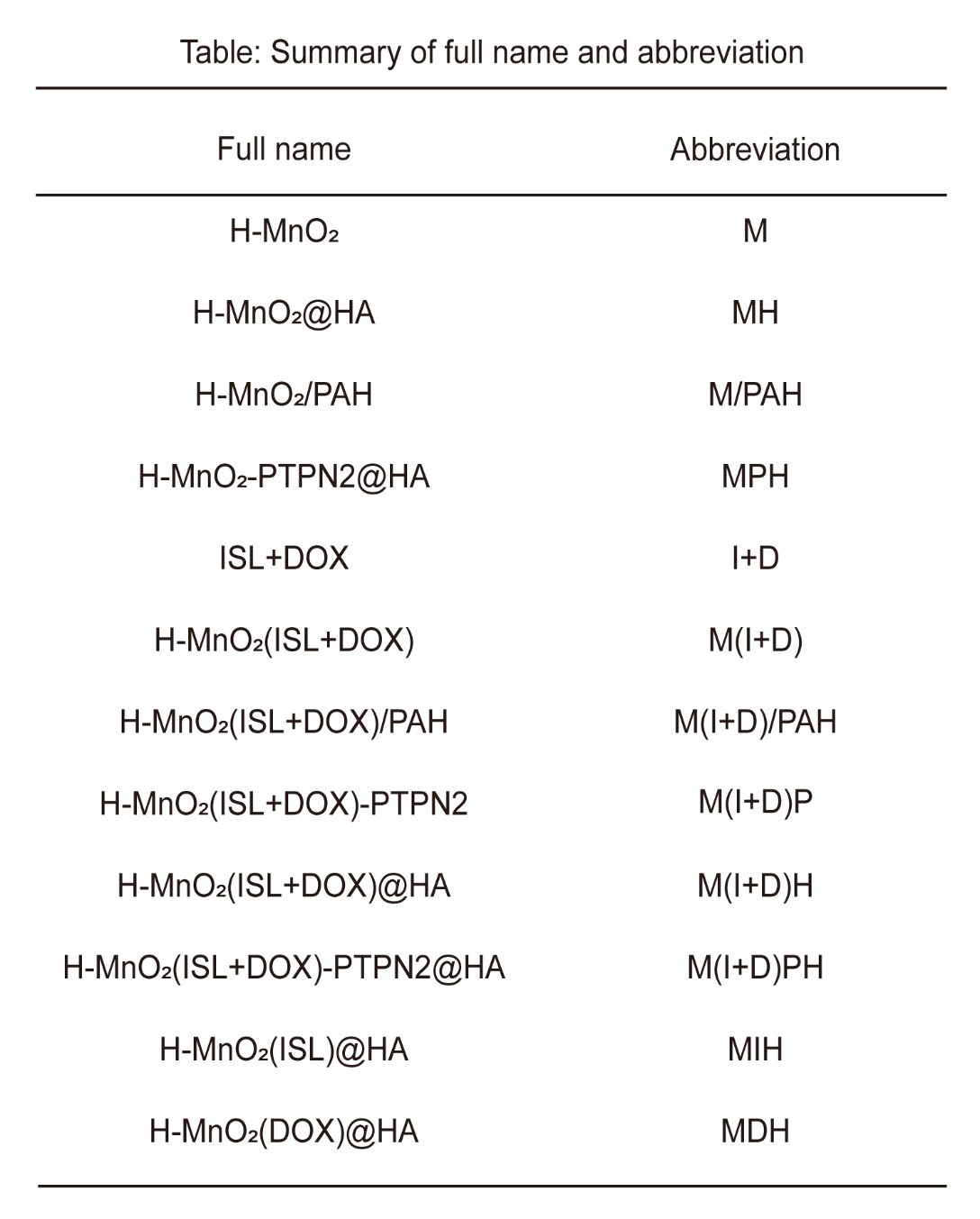


**Table:** Summary of full name and abbreviation.
